# Self-assembled gel tubes, filaments and 3D-printing with *in situ* metal nanoparticle formation and enhanced stem cell growth†

**DOI:** 10.1039/d1sc06062g

**Published:** 2022-01-27

**Authors:** Carmen C. Piras, Alasdair G. Kay, Paul G. Genever, Juliette Fitremann, David K. Smith

**Affiliations:** Department of Chemistry, University of York Heslington York YO10 5DD UK david.smith@york.ac.uk; Department of Biology, University of York Heslington York YO10 5DD UK; IMRCP, UMR 5623, CNRS, Université de Toulouse 118 Route de Narbonne F-31062 Toulouse France

## Abstract

This paper reports simple strategies to fabricate self-assembled artificial tubular and filamentous systems from a low molecular weight gelator (LMWG). In the first strategy, tubular ‘core–shell’ gel structures based on the dibenzylidenesorbitol-based LMWG DBS-CONHNH_2_ were made in combination with the polymer gelator (PG) calcium alginate. In the second approach, gel filaments based on DBS-CONHNH_2_ alone were prepared by wet spinning at elevated concentrations using a ‘solvent-switch’ approach. The higher concentrations used in wet-spinning prevent the need for a supporting PG. Furthermore, this can be extended into a 3D-printing method, with the printed LMWG objects showing excellent stability for at least a week in water. The LMWG retains its unique ability for *in situ* precious metal reduction, yielding Au nanoparticles (AuNPs) within the tubes and filaments when they are exposed to AuCl_3_ solutions. Since the gel filaments have a higher loading of DBS-CONHNH_2_, they can be loaded with significantly more AuNPs. Cytotoxicity and viability studies on human mesenchymal stem cells show that the DBS-CONHNH_2_ and DBS-CONHNH_2_/alginate hybrid gels loaded with AuNPs are biocompatible, with the presence of AuNPs enhancing stem cell metabolism. Taken together, these results indicate that DBS-CONHNH_2_ can be shaped and 3D-printed, and has considerable potential for use in tissue engineering applications.

## Abstract 2

Simple fabrication and 3D-printing methods are used to generate tubes and filaments from self-assembled gels, which can be loaded *in situ* with gold nanoparticles, with the resulting gels encouraging stem cell proliferation.

## Graphical abstract

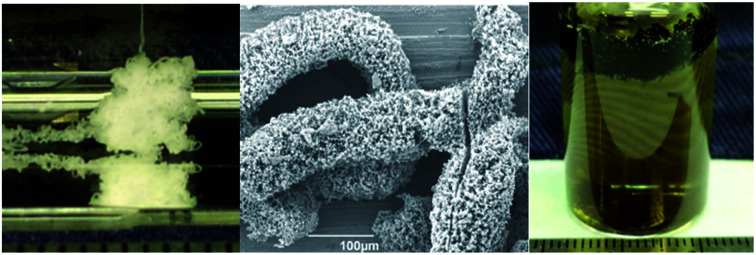

## Introduction

Artificial tubular and filamentous systems with internal fibrillar structuring are attracting growing interest due to their similarity to native human tissues (*e.g.* blood vessels,^1^ nerves,^2^ tendons,^3^ muscles^4^ and bones^5^), which they therefore have potential to replace or help heal. For example, significant efforts have been devoted to the development of biomaterials to treat critically sized bone defects and it has been demonstrated that tubular, porous scaffolds mimicking the native architecture of bone can be highly beneficial in facilitating tissue repair and regeneration.^6^

The fabrication of gels as tubes or filaments can be achieved using a range of techniques, including 3D printing,^7^ wet/electro-spinning,^8^ and microfluidics.^9^ These technologies have seen very rapid expansion in the field of polymer hydrogels. However, their application to low molecular weight gelators (LMWGs) still remains limited. LMWGs are small molecules that can self-assemble in solvents as a result of intermolecular non-covalent interactions giving rise to more complex self-assembled nanostructures.^10^ Compared to polymer gels, the ‘supramolecular gels’ formed from LMWGs are significantly mechanically weaker and, therefore, imposing and retaining a shape is often not easily achievable, thus limiting the range of applications for this class of material.^11^ However, hydrogel scaffolds based on LMWGs have the advantages of higher degradability, stimuli responsiveness and offer greater versatility in terms of chemical modifications. Only a few examples of LMWGs shaped into tubular or filamentous structures have been reported, mostly limited to peptide gelators.^12^ Pioneering research was carried out by Stupp and coworkers, who described the fabrication of string-shaped hydrogels by injection of a peptide solution into salty media.^13^ A similar methodology was adopted by the groups of Hartgerink^14^ and Mihara,^15^ who prepared self-assembled viscoelastic gel strings by injection of a peptide LMWG into a buffered solution or a CaCl_2_ bath. More recently, the use of wet spinning has been described to obtain thin gel filaments from sugar-based LMWGs.^16^

**Fig. 1:**
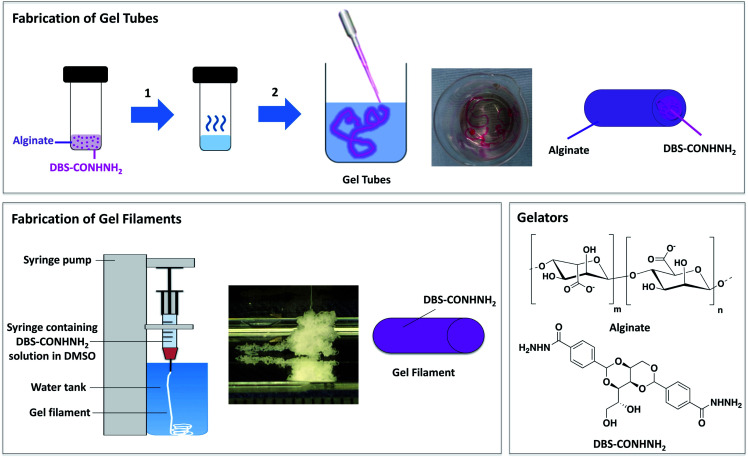
Schematic representation of: (Top) fabrication of DBS-CONHNH_2_/alginate core–shell gel tubes: (1) a mixture of the two gelators (DBS-CONHNH_2_ 0.3% wt/vol and sodium alginate 0.5% wt/vol) is heated until complete dissolution; (2) the hot solution is then added as a thin stream to a CaCl_2_ bath (5.0% wt/vol). Photograph of gel tubes stained with a dye and schematic of gel tube composition. (Bottom left) Fabrication of DBS-CONHNH_2_ gel filaments by wet spinning, photograph of the filament produced and schematic of gel filament composition. (Bottom right) Chemical structures of gelators: alginate (PG) and DBS-CONHNH_2_ (LMWG).

Using a multicomponent approach, combining a LMWG with a polymer gelator (PG), is one way of overcoming the issue of mechanical stability, enabling effective harnessing of LMWG behavior and responsiveness.^17^ In this regard, we recently established a versatile multicomponent gel formulation based on the LMWG 1,3:2,4-di-(4-acylhydrazide)-benzylidenesorbitol (DBS-CONHNH_2_) and the PG calcium alginate (Fig. 1).^18^ By temporal control over the gelation process, we could direct the spatial arrangement of the two gelators within the hybrid gels, forming core–shell or interpenetrated network gel beads.^18^ It was also briefly demonstrated that this fabrication method can be easily modified to obtain core–shell DBS-CONHNH_2_/alginate gel tubes.

This paper explores simple, cost-effective, procedures to fabricate self-assembled LMWG tubes and filaments based on DBS-CONHNH_2_ alone or in combination with the PG calcium alginate (Fig. 1 and 3). To the best of our knowledge, this is one of the few examples of an LMWG tubular system and a rare example of an LMWG gel filament.^13–16^ A unique characteristic of our LMWG (DBS-CONHNH_2_) is that it enables the *in situ* formation of gold nanoparticles (AuNPs) *via* the reduction of gold salts.^19^ To harness this unique property, the gels were loaded with AuNPs, characterised, and biological studies were carried out using human mesenchymal stem cells. Calcium rich polysaccharide gels such as calcium alginate are extracellular matrix mimetics that are potentially well-suited for bone growth.^20^ Furthermore, AuNPs have also previously been demonstrated to be biocompatible and to promote osteogenic differentiation.^21^ Therefore we reason that our tubular and fibrillar gels could have longer-term promise in bone tissue engineering.

## Results and discussion

### DBS-CONHNH_2_/alginate (LMWG/PG) hybrid gel tubes

**Fig. 2:**
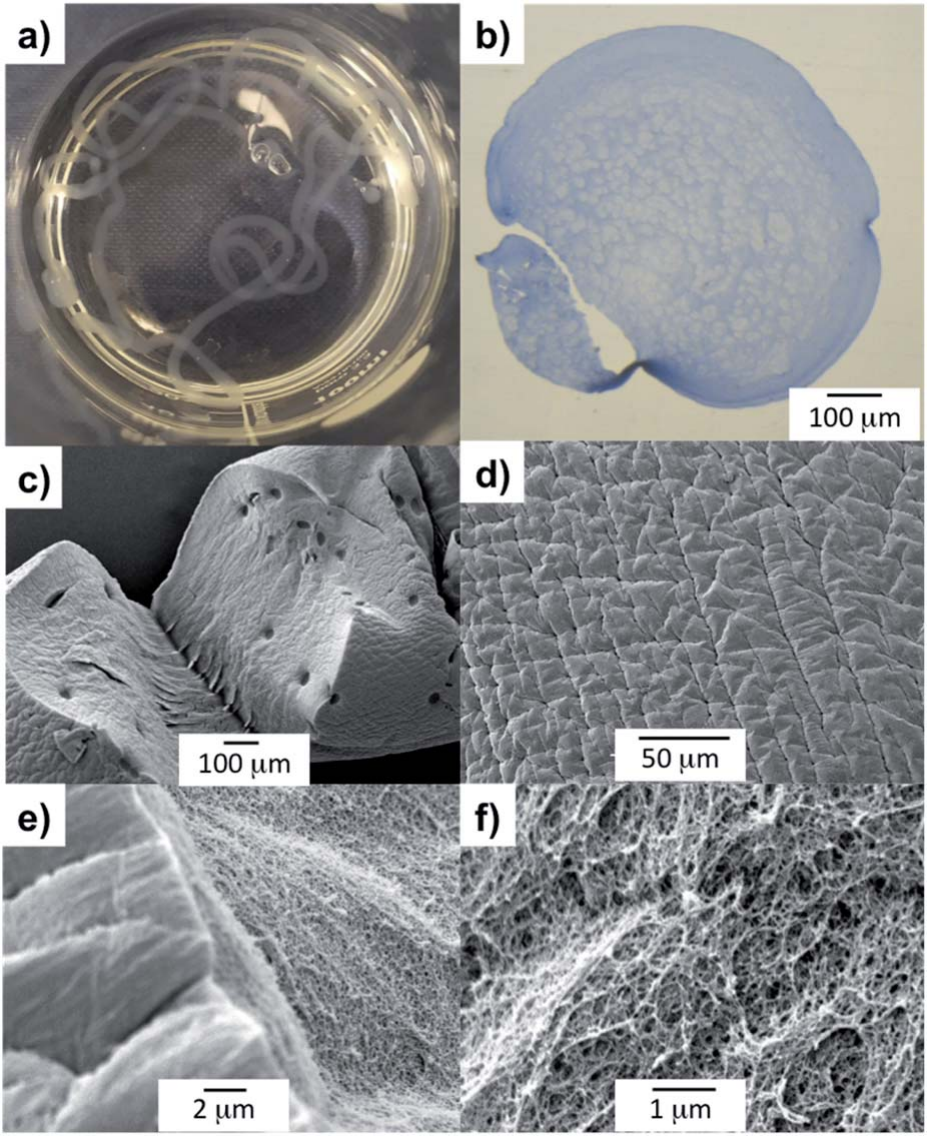
DBS-CONHNH_2_/alginate gel tubes images. (a) Photographic image of the hybrid gel tube in CaCl_2_. (b) Optical microscopy image of the gel tube cross-section embedded in resin and stained with toluidine blue; scale bar: 100 μm. (c and d) SEM images of the gel tube surface; scale bars 100 μm (c) and 50 μm (d). (e and f) SEM images of the gel tube cross-section; scale bars 2 μm (e) and 1 μm (f).

DBS-CONHNH_2_ was synthesized in good yield using our previously reported method.^22^ This LMWG forms gels by heat-cool cycle (0.28–0.40% wt/vol). Low-viscosity alginate is a commercially-available polysaccharide that forms hydrogels upon cross-linking with calcium ions.^23^ As briefly described in our previous work,^18*a*^ DBS-CONHNH_2_/alginate gel tubes were obtained using a simple methodology (Fig. 1). In summary, DBS-CONHNH_2_ (0.3% wt/vol) was combined with sodium alginate (0.5% wt/vol) in water (1 mL), to give a suspension, which was sonicated, heated until complete dissolution, and then injected into a CaCl_2_ bath (5.0% wt/vol) as a thin stream using a glass pipette. Long gel tubes, with a 1.0–1.5 mm diameter and a length of a few cm, which varied depending on the volume taken up with the glass pipette, were immediately formed (Fig. 1, 2a and S1†). The hybrid gel tubes could also be prepared using a higher alginate concentration (0.75, 1.0% wt/vol), whereas a lower alginate concentration (0.3% wt/vol) gave a less homogenous stream and very weak and irregular gel tubes.

Going beyond our previous work,^18*a*^ we then characterised these tubes in some detail. To verify the spatial arrangement of the two gelators, we performed optical microscopy on a cross-section of the gel cylinders embedded in resin and stained with toluidine blue (Fig. 2b). The collected images clearly show a difference between the interior of the cross-section (*ca.* 0.75–0.95 mm), which appears quite porous compared to the outer shell (*ca.* 0.1–0.2 mm), which displays a homogeneous texture. This exterior texture was also observed for the gel filaments prepared using alginate alone (Fig. S7†). These observations suggest that the DBS-CONHNH_2_ self-assembled network forms the core of the gel tubes and calcium alginate forms the surrounding tubular shell.

**Fig. 3:**
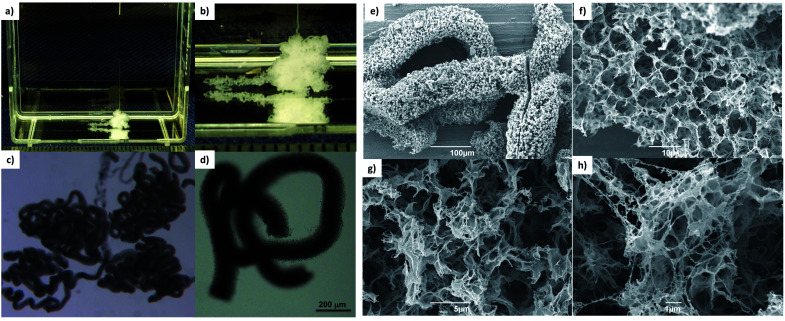
(a and b) photographic images of DBS-CONHNH_2_ gel filament (3.0% wt/vol) adjacent to a ruler (scale in cm; 23 G needle, 3.4 μL min^−1^ flow rate); (c and d) optical microscopy images of DBS-CONHNH_2_ gel filament (scale bar: 200 μm); (e–h) SEM images of DBS-CONHNH_2_ gel filament (scale bars from e-h 100, 10, 5 and 1 μm).

SEM analysis showed a wrinkled surface (Fig. 2c, d and S11†) and a densely packed nanofibrillar network in the hybrid gel tube interior (Fig. 2e and f). The images are consistent with what was previously observed for DBS-CONHNH_2_/alginate core–shell gel beads^18*a*^ and confirms that the incorporated LMWG was in its self-assembled state. This was further confirmed by ^1^H NMR of a small portion (*ca.* 1 cm) of the hybrid gel tube prepared using D_2_O instead of water and transferred into a NMR tube containing D_2_O (0.5 mL) and DMSO (1.4 μL) as an internal standard (ESI,† Section S2.2.1). If DBS-CONHNH_2_ was not in its self-assembled state, the percentage of mobile gelator could be calculated by comparison of the integral peaks of the DBS-CONHNH_2_ aromatic peaks (*δ* = 7.53 and 7.83) to that of the methyl groups of DMSO (*δ* = 2.09 ppm). The ^1^H NMR spectrum showed no signals for the LMWG (or indeed alginate), thus confirming that both gelators were fully self-assembled into the ‘solid-like’ state within the gel tube (Fig. S2†).

By dissolving the dried DBS-CONHNH_2_/alginate core–shell gel tubes in DMSO-d6 in the presence of MeCN as an internal standard, and performing NMR spectroscopy, we were able to quantify the exact amount of DBS-CONHNH_2_ incorporated (ESI Section 2.2.2†). A gel tube prepared with 1 mL of water using 0.3% wt/vol of LMWG (6.32 μmoles) and 0.5% wt/vol of alginate, incorporates *ca.* 6.30 μmoles of DBS-CONHNH_2_, which corresponds to >99.5% of the loaded LMWG (Fig. S5†). This confirms the efficiency of the fabrication method.

The system described is consistent with a model in which the calcium alginate PG rapidly forms as the solution is added into the calcium chloride bath and effectively acts as a ‘tubular mould’ for the thermally-induced self-assembly of the LMWG on cooling, which otherwise would not be able to retain its shape. This is also consistent with a degree of phase separation between PG and LMWG. The presence of some supramolecular interactions between the two gel components is, however, supported by IR spectroscopy of the DBS-CONHNH_2_/alginate filament xerogels, which clearly shows broadened O–H (3311 cm^−1^) and N–H (3187 cm^−1^) stretching bands of DBS-CONHNH_2_ in the presence of the PG (Fig. S6†).

We previously demonstrated that in such hybrid DBS-CONHNH_2_/alginate gels, the percentage of PG could be varied, thus changing the robustness of the resulting material.^18*a*^ By contrast, the concentration of LMWG cannot be as easily modified – below 0.28% wt/vol loading, the DBS-CONHNH_2_ does not form self-supporting hydrogels and above 0.40% wt/vol it does not dissolve completely when heating is applied to trigger gelation. Therefore, the DBS-CONHNH_2_ concentration range that can be employed to obtain hybrid gel tubes is limited to 0.28–0.40% wt/vol. Such low concentrations can be advantageous to prepare high water content materials (>99%) that closely mimic extracellular matrix, however, a higher LMWG concentration could be beneficial when gel function is correlated to this parameter. For this reason, we were also interested in exploring the fabrication of self-assembled DBS-CONHNH_2_ gel filaments at a higher LMWG concentration (see below). We reasoned that if higher concentration gel objects could be obtained from DBS-CONHNH_2_, they may become self-supporting even in the absence of the calcium alginate PG.

### DBS-CONHNH_2_ gel filaments by wet spinning

DBS-CONHNH_2_ gel filaments were prepared by wet spinning. This method has been extensively studied for polymers^24^ but has only very recently also been applied to LMWGs.^16^ It is a type of solution spinning, where a solid gelator is dissolved in a good solvent and then extruded into a coagulant solution with which the gelator self-assembles when in contact. Gel fibres rapidly formed *via* self-assembly following the mutual diffusion of solvent and non-solvent.

DBS-CONHNH_2_ gel filaments were fabricated as follows. The LMWG was dissolved in anhydrous DMSO. The use of this good solvent facilitates very high loadings of the gelator (1.5, 3.0 or 4.5% wt/vol). The resulting solution was then transferred to a 1 mL syringe and slowly released into a water bath through a blunt tip needle at a known flow rate (Fig. 1 and 3).

To identify optimal conditions for the formation of the gel filaments, we used different LMWG concentrations, needle diameters and flow rates. Uniform gel filaments with 80–185 μm diameter were obtained at the slowest flow rates (3.4 and 6.7 μL min^−1^) using a 150 or 330 μm inner diameter needle (respectively 30 G and 23 G blunt tip needle; Fig. 1, 3a–d, S14 and ESI video†). Larger needle diameters (610, 840 and 1370 μm) released the gelator too rapidly and led to clogging of the needle (Fig. S15†) or less-controlled gelation at the bottom of the tank (Fig. S16†). These observations are summarised diagrammatically in the ESI (Fig. S17)†.

We observed that the gel filaments were quite delicate and prone to breakage when handled. To check the stability of gel filaments over time, we performed optical microscopy on a freshly made sample and a sample after storage for 30 days in water (Fig. S18–S22†). Pleasingly, no significant variations were observed after this time, thus showing that although they are very delicate, the gel filaments are stable in water for at least a month.

To gain insight into the fibrillar network of the DBS-CONHNH_2_ gel filaments at the nanoscale, we performed TEM and SEM analysis. Both techniques showed the presence of an intricate fibrillar network on the surface and the interior of the filaments (Fig. 3e–h, S23 and S25–27†). Interestingly, compared to the DBS-CONHNH_2_/alginate core–shell gel strings, the surface of these self-assembled tubular structures appear to be ‘sponge-like’ and much more ‘porous’. Conversely, the surface of the DBS-CONHNH_2_/alginate hybrid gel tubes displayed a more compact texture, consistent with the presence of the supporting PG shell/mould around the fibrillar LMWG network.

It is important to note that the wet spinning process, which relies on the use of a co-solvent (DMSO) to mediate gelator solubility, probably triggers a different self-assembly process compared to the heat-cool cycle applied to induce gelation in pure water. Indeed, the fibre organisation and the fibre width of the bulk gel prepared by a heat-cool cycle (Fig. S24 and S28†) appears quite different by TEM and SEM compared to the gel filaments produced by wet spinning (Fig. 3e–h, S23 and S25–27†). However, the appearance of the DBS-CONHNH_2_ gel network may be somewhat impacted by drying effects. Subtle differences in self-assembly are consistent with previously reported observations of the wet spinning process applied to LMWGs inducing different fibre arrangements and sizes.^16*a*^

After formation of the DBS-CONHNH_2_ gel filaments, we reasoned that the DMSO used to facilitate their assembly would exchange with water during standing/washing. To quantify this, we performed an NMR experiment in which filaments at different loading concentrations were extruded in 50 μL of DMSO. After gently washing the resulting filaments twice in D_2_O and drying, the solid material was then placed in D_2_O with an internal standard (CH_3_CN) and a ^1^H NMR spectrum recorded (Fig. S29–S31†). In this way, the residual DMSO could be quantified (Table S2†). At 1.5% loading, 0.015 μL of DMSO remained. This increased to 0.33 μL at 3.0% loading and to 0.55 μL at 4.5% loading. This means that at 4.5% LMWG loading, 98.9% of the DMSO has been removed by washing, rising to 99.9%+ at 1.5% LMWG loading. We can therefore be confident that the gel filaments have insignificant quantities of DMSO within them, that should not be problematic for cell culture.

The exact amount of DBS-CONHNH_2_ incorporated into the gel filaments prepared by wet spinning using 1.5, 3.0 and 4.5% wt/vol concentrations of LMWG (23 G blunt tip needle and 3.4 μL min^−1^ flow rate), was also quantified by ^1^H NMR of the dried filaments (50 μL), fully dissolved in DMSO-d6 in the presence of CH_3_CN as an internal standard. By comparison of the integrals of the aromatic signals of the LMWG to that of CH_3_CN, we were able to estimate that >95% of loaded LMWG was incorporated into the gel filaments at all of the different concentrations (Fig. S32–S34†). This indicates a highly efficient wet-spinning process in which the LMWG is effectively all ‘printed’ into filament form.

### 3D printing of DBS-CONHNH_2_ gel filaments

**Fig. 4:**
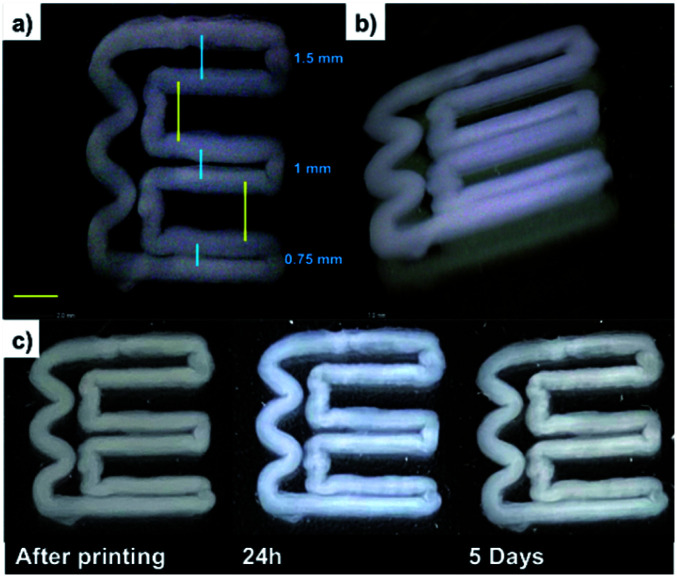
(a and b) DinoLite microscopy images of DBS-CONHNH_2_ 3D printed gel (3.0% wt/vol, 10 layers, yellow scale bar. 2 mm); (c) photographic images of DBS-CONHNH_2_ 3D printed gel over time.

To investigate whether the DBS-CONHNH_2_ gel filaments prepared by wet spinning could be extruded into well-defined patterns over several layers, we further adapted this method to 3D printing. Due to the poor mechanical properties of LMWGs in comparison to polymers, 3D-printing still remains relatively unexplored for LMWGs.^16,25^ 3D-printing of DBS-CONHNH_2_ was performed by coupling the syringe pump with a drawing robot moving the nozzle in *x* and *y* directions, with movement in the *z* direction being achieved with a z-micrometric platform. A DBS-CONHNH_2_ solution in DMSO (3.0% wt/vol) was injected in a static water bath in a polystyrene Petri dish by moving the nozzle to obtain a geometric pattern (Fig. 4a–b, S36 and ESI video†).

The resulting 3D-printed architecture was based on the deposition of several layers, forming a construct that was stable in water for at least 5 days (Fig. 4c). Indeed, even after 8 days, no visible change was observed. This result is remarkable considering that a previously reported LMWG that was 3D-printed in this way (*N*-heptyl-d-galactonamide, GalC7) was stable for less than 24 hours (Figs S36–S38†). This is due to the difference of solubility between the two gelators, with the lower solubility of DBS-CONHNH_2_ in water giving its self-assembled 3D-printed structures much greater stability against morphological changes. Since the stability of 3D printed constructs in water is a fundamental parameter that can facilitate their successful use in cell culture applications, this result clearly demonstrates the potential for DBS-CONHNH_2_ to be shaped and structured for tissue engineering applications. The shape fidelity was assessed by printing a pattern including lines spaced with decreasing distances (‘filament fusion test’), right angles and curves. Ten layers were printed. A distance of 1.5 mm was left between the top two lines of the ‘*E*’, 1.0 mm between the middle lines, and 0.75 mm between the bottom two lines (Fig. 4a). The gel sets fast enough in contact with water to avoid too much spreading of the liquid ink, resulting in two distinct lines even at less than 0.75 mm distance. The right angles and waves were also well-defined. This result is very good in terms of shape fidelity compared with what is usually observed with this technique of ‘direct ink writing’ 3D printing. We anticipate that this advantage will apply to even more complex shapes, which are under investigation in our laboratories.

We note that the wet spinning technique can, in principle, be easily applied to other LMWGs. It is important for optimal conditions to be met (*e.g.* solubility in DMSO, and rapid gelation to allow the deposition of well-resolved patterns). Key parameters such as flow rate, needle diameter and temperature will then have to be optimized in each case.^16^

### Incorporation of AuNPs into gel tubes and filaments

To demonstrate that the unique characteristics of DBS-CONHNH_2_ were translated into the tubes and filaments described above, we decided to exploit the LMWG's unique ability to reduce metals to induce the *in situ* formation of AuNPs.^19^ There has been considerable interest in the development of LMWG hydrogels that can spontaneously generate AuNPs.^26^ In this case, we reasoned that the fabrication of gel filaments incorporating potentially conductive AuNPs could ultimately be very interesting in biology for bone tissue engineering.^21*c*,27^ Gels incorporating AuNPs can potentially be used for cell electrical stimulation, facilitating bone repair.^28^ It has also been demonstrated AuNPs can enhance mesenchymal cell proliferation and osteogenic differentiation.^29^

**Fig. 5:**
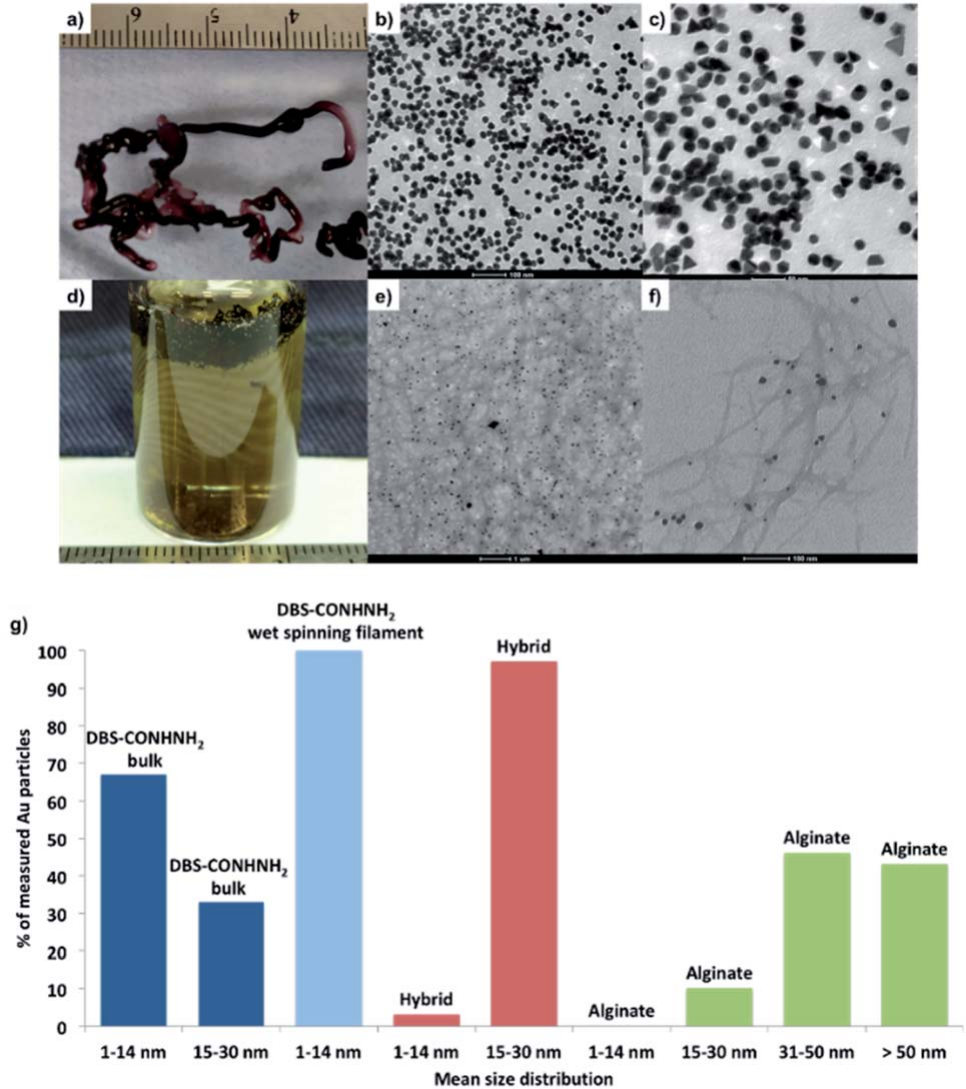
(a–c) Photographic and TEM images of DBS-CONHNH_2_/alginate hybrid gel filament loaded with Au NPs (Scale bars b and c: 100 and 50 nm). (d–f) Photographic and TEM images of DBS-CONHNH_2_ wet spinning gel filament loaded with Au NPs (Scale bars e and f: 1 μm and 100 nm). (g) Graph of Au NPs size distribution in DBS-COHNH_2_ bulk gel (dark blue), DBS-CONHNH_2_ wet spinning gel filament (light blue), DBS-CONHNH_2_/alginate hybrid gel filament (red) and alginate gel filament (green). The percentages refer to the percentage of total AuNPs in the sample that were within each size band.

To induce *in situ* formation of AuNPs, we simply immersed the gels in aqueous AuCl_3_ for 24 hours. A colour change from white to purple was observed almost immediately upon the reduction of Au(iii) to Au(0) (Fig. 5a and d and S39†). To confirm the formation of the AuNPs and measure their size, we performed TEM analysis on the different types of gels, which showed uniformly distributed NPs within the DBS-CONHNH_2_ bulk gel (mostly 1–14 nm diameter NPs, some 15–30 nm NPs; Fig. 5g and S40†), the DBS-CONHNH_2_/alginate hybrid gel tubes (15–30 nm diameter NPs; Fig. 5a–c, g and S41†) and the DBS-CONHNH_2_ wet-spun gel filaments (1–14 nm diameter NPs; Fig. 5d–g and S42†). As a control, we also analysed alginate-only gel tubes (0.8% wt/vol; Fig. 5g and S38†) produced by extruding alginic acid (0.8% wt/vol) into an aqueous CaCl_2_ bath. Metal NPs were also visible in these gels; however, they were quite irregular and tended to aggregate into larger clusters >30 nm in diameter (Fig. 5g and S38†). Reduction of Au(iii) to AuNPs is much less efficient in this case, as demonstrated by the much smaller number of NPs and the lack of a visible colour change of the alginate gels (Fig. S39 and S43†). This clearly indicates the key role of the acyl hydrazide functional groups of the DBS-CONHNH_2_ LMWG in helping mediate the *in situ* reduction process.^19^

To quantify the maximum amount of Au that could be incorporated into these materials, we immersed the gels in a concentrated AuCl_3_ solution (20 mM, 1 mL) for 24 hours and then measured the UV absorbance of the supernatant containing the non-incorporated Au(iii). For the hybrid gel tubes, the maximum Au(iii) uptake was 15.7 μmol of Au/mL of gel (Table S3 and Fig. S44†), which was more than double than the alginate-only tube (7.20 μmol of Au/mL of gel). The same gels prepared in sample vials gave similar results, with the DBS-hybrid gel performing better (14.7 μmol of Au/mL of gel) than the alginate-only gel (7.90 μmol of Au/mL of gel). This indicates that the gel tubes behave similarly to the gels in vials in terms of Au uptake. Pleasingly, the DBS-CONHNH_2_ retains its reducing power when incorporated into the hybrid gels, giving similar metal uptake to that of the DBS-CONHNH_2_ gel alone prepared in vials (16.5 μmol of Au/mL of gel; Table S3 and Fig S44†).

The rate of Au uptake was also studied using a lower Au concentration (2.5 mM, 2 mL). Although 100% uptake was reached after 24 hours by the DBS-CONHNH_2_ gel, the hybrid gel in vials and the DBS-CONHNH_2_ gel tube (Table S4 and Fig S45†), the process was slightly faster for the tube (*ca.* 66%) after 3 hours, than the DBS-CONHNH_2_/alginate gel prepared in a sample vial (*ca.* 53%), possibly showing an advantage of the larger surface area of the gel tube compared to the corresponding gel prepared in a sample vial.

We hypothesised that a higher LMWG concentration would significantly increase Au uptake, given the mechanism of uptake relies on acyl hydrazide mediated reduction of Au(iii) to Au(0). We therefore studied the maximum uptake of the gel filaments prepared by wet spinning using 1.5, 3.0 and 4.5% wt/vol concentrations of DBS-CONHNH_2_ (23 G blunt tip needle and 3.4 μL min^−1^ flow rate). As expected, due to the higher LMWG concentration, the performance of the DBS-CONHNH_2_ gel filaments was outstanding compared to the DBS-CONHNH_2_ gel prepared in sample vials and the hybrid gel either in sample vials or tubes (0.3% wt/vol LMWG), with *ca.* 10–20 times greater uptake being exhibited by the filaments (in-line with the higher LMWG concentration). An increasing amount of Au(iii) was taken up at increasing LMWG concentrations (respectively 127.0, 190.6 and 298.6 μmol of Au/mL of gel; Table S5 and Fig. S46†).

The influence of the AuNPs on the thermal stability and mechanical properties of the gels, were studied by gel–sol transition temperature (*T*_gel_) determination and parallel plate oscillatory rheology. For practical reasons, these experiments were conducted on the different gels prepared in sample vials. *T*_gel_ determination was performed using a simple tube inversion method. The *T*_gel_ of the DBS-CONHNH_2_ alone (0.4% wt/vol) is 86 °C, but in the presence of increasing Au loading (5, 10 and 20 μmol of AuCl_3_ added on top of the gel), the *T*_gel_ increased to >100 °C (ESI Table S5†). This is consistent with our previously reported observations^19^ and may be due to cross-linking and/or reinforcement of the gel fibres in the presence of the AuNPs. The DBS-CONHNH_2_/alginate hybrid gel (0.3% wt/vol of DBS-CONHNH_2_ and 0.5% wt/vol of alginate) and the alginate gel (0.8% wt/vol) exposed to the same Au loadings showed a *T*_gel_ of >100 °C (Table S6†) in each case, confirming that the presence of AuNPs does not adversely affect the thermal stability of the gels across the analysed temperature range (25–100 °C).

Oscillatory rheology gave us insight into the mechanical properties of the Au-loaded gels. The DBS-CONHNH_2_ hydrogel (0.4% wt/vol) has an elastic modulus (*G*′) of 786 Pa, which, in the presence of increasing Au loadings (5 and 10 μmol of AuCl_3_ added on top of the gels), was not significantly affected (*G*′ = 758 Pa and 634 Pa, respectively; Table S7 and Fig. S45–S48†). It is therefore clear that the gel maintains its stability in the presence of AuNPs. The DBS-CONHNH_2_/alginate hybrid gel (0.3% wt/vol of DBS-CONHNH_2_ and 0.5% wt/vol of alginate; *G*′ = 8260 Pa, Table S7 and Fig. S50†) showed a similar elastic modulus with 5 μmol of AuCl_3_ added on top of the gel (*G*′ = 8870 Pa, Table S7 and Fig. S54†) and a higher *G*′ value in the presence of 10 μmol of AuCl_3_ added on top of the gel (*G*′ = 16 100 Pa, Table S7 and Fig. S52†). A similar effect was observed for the alginate gel (0.8% wt/vol, Table S7 and Fig. S56–S58†). This is probably due to some mechanical reinforcement of these hybrid gels by the AuNPs. In general terms, it is well-known that metal nanoparticles can mechanically reinforce gels.^30^ A slight increase in *G*′ and *G*′′ is already observed at lower AuNPs loadings, however, this effect is quite significant at higher loadings, due to the higher amount of AuNPs incorporated.

### Biological results

**Fig. 6:**
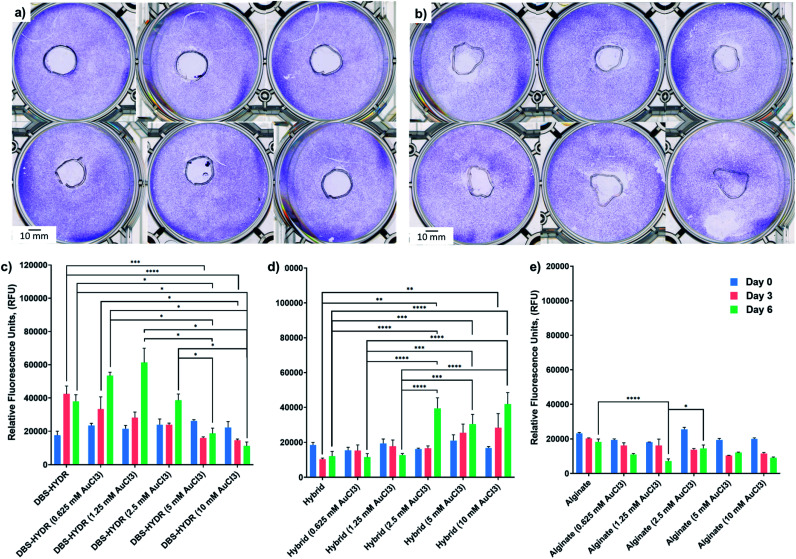
Cytotoxicity and viability assay results. (a and b) Scanned images of crystal violet stained cells seeded around DBS-CONHNH_2_/alginate hybrid gels (a) and alginate gels (b) loaded with 1 (upper rows) and 10 (lower rows) μmol of AuCl_3_/mL of gel. (c–e) Alamar blue assay results for gels loaded with different AuCl_3_ concentrations. *N* = 6, mean reported, error bars represent standard error, DBS-HYDR = DBS-CONHNH_2_; *p < 0.05, **p < 0.01; ***p < 0.001; ****p < 0.0001 (one-way ANOVA).

To test the potential applicability of our gels in biology, we carried out preliminary cytotoxicity and cell viability studies using human mesenchymal stem cell line (Y201).^31^ Cytotoxicity testing was performed on the gels without AuNPs and the gels loaded with 1 and 10 μmol of AuCl_3_/mL of gel. The gels were prepared in a 48-well plate and placed in the middle of a 6-well plate, where the cells were then seeded. We note that the DBS-CONHNH_2_ gels (with/without NPs) are fragile and could not be transferred without breakage from one plate to another. Therefore, the DBS-CONHNH_2_ gels were prepared directly in the 6 well plate by heat-cool cycle using bottomless vials. After 48 hours, the cells were fixed and stained with crystal violet and the plates were scanned. If the gels were toxic, we expected to see a ring without cells around the gels (a sort of ‘zone of inhibition’ of cell growth). Pleasingly, none of the gels (with/without NPs) showed a ‘zone of inhibition’ after 48 hours (Fig. 6a and b and S59†); therefore we decided to perform a viability test on cells seeded on top of the gels.

We prepared the gels in non-adherent 96-well plates and soaked them in AuCl_3_ for 72 hours 0.625, 1.25, 2.5, 5 and 10 μmol of AuCl_3_/mL of gel. After this time, the gels were washed multiple times with DMEM (10% FBS, 1% P/S) and the cells (25000/well) were then seeded on top of the gels. Cell viability was measured at day 0, 3 and 6 by the Alamar blue viability assay, which measures the metabolic activity of the cells.^32^ The obtained results show that the cells were alive and metabolically active for the whole duration of the study (Fig. 5c–e). Interestingly, the DBS-CONHNH_2_ gels loaded with 0.625 and 1.25 μmol of AuCl_3_/mL of gel showed very slightly higher fluorescence at day 6 compared to the gels without AuNPs (Fig. 6c). This suggests that the presence of AuNPs may induce an increase in cell metabolic activity. By contrast, at 5.0 and 10 μmol of AuCl_3_/mL of gel the fluorescence signal decreases significantly at day 6 (Fig. 6c). However, at these higher AuCl_3_ concentrations, the DBS-CONHNH_2_ gels were more fragile and were significantly affected by manipulation over the six days, undergoing breakage and fragmentation. It is important to highlight that, since non-adherent plates were used, the gels were the only adhesion surface available for the cells and, therefore, gel breakage/fragmentation could dramatically impact cell viability. In the case of the DBS-CONHNH_2_-only gels, the removal of broken gel fragments during media changes would be a reason for a lower cell number, due to physical removal of cells adhering to the fragments. The lower detected metabolic activity could therefore be related to gel breakage rather than the presence of a higher concentration of AuNPs – indeed we did visually see some gel damage, especially at higher AuNP loadings.

Supporting this view, the much more robust DBS-CONHNH_2_/alginate hybrid gels loaded with 2.5, 5.0 and 10.0 μmol of AuCl_3_/mL of gel showed a higher fluorescence at day 6 compared to the hybrid gels without AuNPs (Fig. 6d). Furthermore, at the lower AuNP loadings of 0.625 and 1.25 μmol of AuCl_3_/mL of gel the increase in metabolic activity detected in the assay was, in this case, highly statistically significant. Again, this indicates that the presence of AuNPs in these gels may have a beneficial effect on the cell metabolic activity, which can be related to a higher number of cells, with the greater robustness of the hybrid gel compared with DBS-CONHNH_2_ alone making this effect more significant in the assay. These observations of the positive impact of AuNPs on cell proliferation are in-line with previously reported studies.^29*c*,33^

Alginate-only gels did not show any increase in metabolic activity over time for the duration of the study (Fig. 6e). Neither did the presence of Au appear to have any beneficial effect. As described above AuNP formation in these gels was not as efficient as for the DBS-CONHNH_2_ and the DBS-CONHNH_2_/alginate gels, so this is consistent with a model in which the presence of AuNPs are indeed responsible for enhancing cell metabolism. Moreover, it has been demonstrated that AuNP cytotoxicity is shape and size dependent.^34^ Therefore, the AuNP clusters (diameter > 50 nm) formed in the alginate gels (Fig. S38†), rather than uniformly distributed smaller nanoparticles, may be less beneficial to cell proliferation over time. It should be noted that the method applied to prepare the gels and load them with cells here is not the most appropriate/optimised for alginate, which could be simply mixed with the cells before cross-linking with Ca^2+^ ions. Nevertheless, this experimental procedure was chosen to compare all of the gels in similar conditions.

These preliminary results therefore demonstrate that the DBS-CONHNH_2_ and the hybrid DBS-CONHNH_2_/alginate gels loaded with AuNPs are compatible with cell growth. Furthermore, it appears that the presence of AuNPs enhances cell metabolism. We therefore suggest that these gels might be very promising materials for biological applications.

## Conclusion

In conclusion, we have reported easy, cost-effective, strategies to obtain LMWG artificial tubular and filamentous constructs. DBS-CONHNH_2_/alginate hybrid gel tubes were fabricated by simple injection of the hot gelator mixture into a CaCl_2_ bath. To the best of our knowledge, this is the only example of core–shell tubular structures based on a LMWG and a PG. This method could be potentially applied to a variety of LMWGs and PGs.

DBS-CONHNH_2_ gel filaments were successfully prepared by wet spinning at different LMWG concentrations. This technique allows self-assembly of the LMWG at high concentrations – this means that the gelator is able to form self-supporting shaped objects, even in the absence of a PG. This is a particularly attractive option when gel function depends on gelator loading, such as here, where the gelator goes on to play an active role in reducing Au(iii) to give AuNPs. Furthermore, this approach demonstrated great potential for 3D printing in multiple layers to give more complex structured architectures, which retained their stability in water for at least five days.

The *in situ* formation of AuNPs spontaneously occurs in both the gel tubes and the gel filaments, when immersed in AuCl_3_ solutions, as a direct consequence of the presence of DBS-CONHNH_2_. The gel filaments could achieve much higher AuNP loading as a result of their higher concentration of DBS-CONHNH_2_. Initial biological screening of the AuNP-loaded gels confirmed that they are biocompatible and furthermore, that the presence of the AuNPs increases the metabolic activity of human mesenchymal stem cells over time.

Taken overall, these results show that our AuNP gel formulations are promising materials for biological tissue engineering applications. Further *in vitro* stem cell studies on the gel tubes and filaments will be carried out in the future to verify cell growth and function, particularly with regard to osteogenesis, and importantly to understand whether shaping the gels can impact on the mode of cell growth. It is worth noting that embedding AuNPs into DBS-CONHNH_2_ gels is also known to make them conductive^19^ – in the future, such constructs could therefore also have impact as high-tech 3D scaffolds for electrical stimulus-responsive cells (*e.g.* stem cells, neurons, muscles).

## Data availability

Relevant experimental data are provided in the ESI.†

## Author contributions

The manuscript was written through contributions of all authors, led by CCP and DKS. Experimental work was performed by CCP, with JF providing specific support in wet-spinning and performing the 3D-printing experiments. AGK performed statistical analysis of stem cell experiments. Experiments were planned by CCP in discussion with PG, JF and DKS. The overall project was managed by DKS.

## Conflicts of interest

There are no conflicts to declare.

## Supplementary Material

SC-013-D1SC06062G-s001
