# Supplementary material for: Self-assembled gel tubes, filaments and 3D-printing with in situ metal nanoparticle formation and enhanced stem cell growth
Source: Chem Sci. 2022 Jan 27;13(7):1972–81. doi: 10.1039/d1sc06062g (PMC8848986; doi:10.1039/d1sc06062g)
Supplement: SC-013-D1SC06062G-s001 [file SC-013-D1SC06062G-s001.pdf]

Electronic Supplementary Material (ESI) for Chemical Science.
This journal is © The Royal Society of Chemistry 2022

Self-assembled gel tubes, filaments and 3D-printing with *in situ* metal nanoparticle formation and enhanced stem cell growth

Carmen C. Piras,[a] Alasdair G. Kay,[b] Paul G. Genever,[b] Juliette Fitremann*[c] and David K. Smith*[a]

a: Department of Chemistry, University of York, Heslington, York, YO10 5DD, UK
b: Department of Biology, University of York, Heslington, York, YO10 5DD, UK
c: Laboratoire des IMRCP, Université Paul Sabatier, 118 Route de Narbonne, Toulouse, 31062 , France
david.smith@york.ac.uk

SUPPORTING INFORMATION

S1 General experimental methods

S2 Preparation and characterisation of DBS-CONHNH$_2$/alginate core-shell gel filaments
S2.1 Gel preparation
S2.2 NMR assays
S2.3 Infrared (IR) spectroscopy
S2.4 Optical microscopy
S2.5 Scanning Electron Microscopy (SEM)

S3 Preparation and characterisation of DBS-CONHNH$_2$ gel filaments by wet spinning
S3.1 Gel preparation
S3.2 Optical microscopy
S3.3 Transmission and Scanning Electron Microscopy (TEM and SEM)
S3.4 NMR assays
S3.5 Infrared (IR) spectroscopy
S3.6 3D printing

S4 Preparation and characterisation of DBS-CONHNH$_2$, DBS-CONHNH$_2$/alginate and alginate gels loaded with Au nanoparticles (NPs)
S4.1 *In situ* formation of Au NPs
S4.2 Transmission Electron Microscopy (TEM)
S4.3 Uptake of Au(III)
S4.4 Infrared (IR) spectroscopy
S4.5 Thermal stability studies
S4.6 Rheology

S5 Biological studies
S5.1 Cytotoxicity assays
S5.2 Viability assay

S6 References

S1 General Experimental Methods

All compounds used in synthesis and analysis were purchased from standard commercial suppliers and used as received. The alginate employed in all the experiments was bought from Sigma Aldrich as sodium salt (2% viscosity). The synthesis of DBS-CONHNH$_2$ was performed in good yields applying previously reported methods.[1, 2] The preparation of DBS-CONHNH$_2$ filaments by wet spinning was performed using an A-99 Razel Syringe Pump. ^{1}H NMR spectra were recorded using a Jeol 400 spectrometer (^{1}H 400 MHz). Samples were prepared in DMSO-d$_6$ or D$_2$O and chemical shifts (δ) are reported in parts per million (ppm). IR spectra of xerogels were recorded on a PerkinElmer Spectrum Two FT-IR spectrometer. Optical microscopy images were obtained using a Zeiss Axiocam camera on a Zeiss stereo microscope. SEM images were taken using a JEOL JSM-7600F field emission SEM. TEM images were obtained on a FEI Tecnai 12 G^2 fitted with a CCD camera. Au nanoparticles diameters were measured using the *ImageJ* software. T_{gel} values were obtained using a high precision thermoregulated oil bath using the tube inversion method and were recorded in triplicate. Thermal gravimetric analysis (TGA) was performed on a Simultaneous Thermal Analyser (PL Thermal Sciences STA 625). Rheology was measured on a Malvern Instruments Kinexus Pro+ Rheometer fitted with a 20 mm parallel plate geometry. Plates for the cytotoxicity assay were imaged with an Epson PhotoScanner. Fluorescence measurements for the cell viability assay were performed using a BMG Labtech Clariostar Plate Reader.

S2 Preparation and characterisation of DBS-CONHNH$_2$/alginate core-shell gel tubes

S2.1 Gel preparation

S2.1.1 DBS-CONHNH$_2$/alginate core-shell gel tubes. DBS-CONHNH$_2$ (0.3% wt/vol) and sodium alginate (0.5% wt/vol) were suspended in water (1 mL) and sonicated to help the dispersion of the solid particles. The resulting suspension was heated until complete dissolution of the DBS-CONHNH$_2$. The hot solution was then added as a thin stream to a CaCl$_2$ solution (5.0% wt/vol, c.a. 50 mL), resulting in a long gel tube. The gel tube was collected by filtration and washed with water multiple times.

S2.1.2 Alginate gel tubes. Alginate gel tubes were prepared by adding an aqueous alginate solution (0.8% wt/vol) to a CaCl$_2$ solution (5.0% wt/vol, c.a. 50 mL) as a thin stream. The gel filaments were collected by filtration and washed with water multiple times.

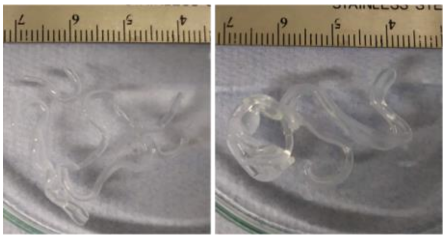

Figure S1. Photographic images of DBS-CONHNH$_2$/alginate core-shell gel tube (0.3% wt/vol DBS-CONHNH$_2$ and 0.5% wt/vol alginate, left) and alginate gel filament (0.8% wt/vol, right).

S2.2 NMR assays

^{1}H NMR was employed to confirm that the DBS-CONHNH$_2$ incorporated into the DBS-CONHNH$_2$/alginate gel tubes was in self-assembled state and to quantify the exact amount of LMWG loaded into a gel tube.

S2.2.1 NMR verification of the self-assembled state of DBS-CONHNH$_2$ incorporated into the DBS-CONHNH$_2$/alginate gel tubes. The gel tube used for this experiment was prepared by combining DBS-CONHNH$_2$ (0.3 % wt/vol) and alginate (0.5 % wt/vol) as described in Section S2.1. A portion of the gel tube (c.a. 1 cm) was transferred into a NMR tube. D_2O (0.5 mL) and anhydrous DMSO (1.4 μL) were then added. The ^{1}H NMR spectrum was recorded and the amount of mobile components was calculated by comparison of the integrals of relevant peaks (DBS-CONHNH$_2$ aromatic peaks δ = 7.53 and 7.83 ppm; Fig. S2) to that of DMSO (δ = 2.50 ppm). As a reference we report below the ^{1}H NMR of solid DBS-CONHNH$_2$ (Fig. Sxx) and alginate (Fig. Sxx).

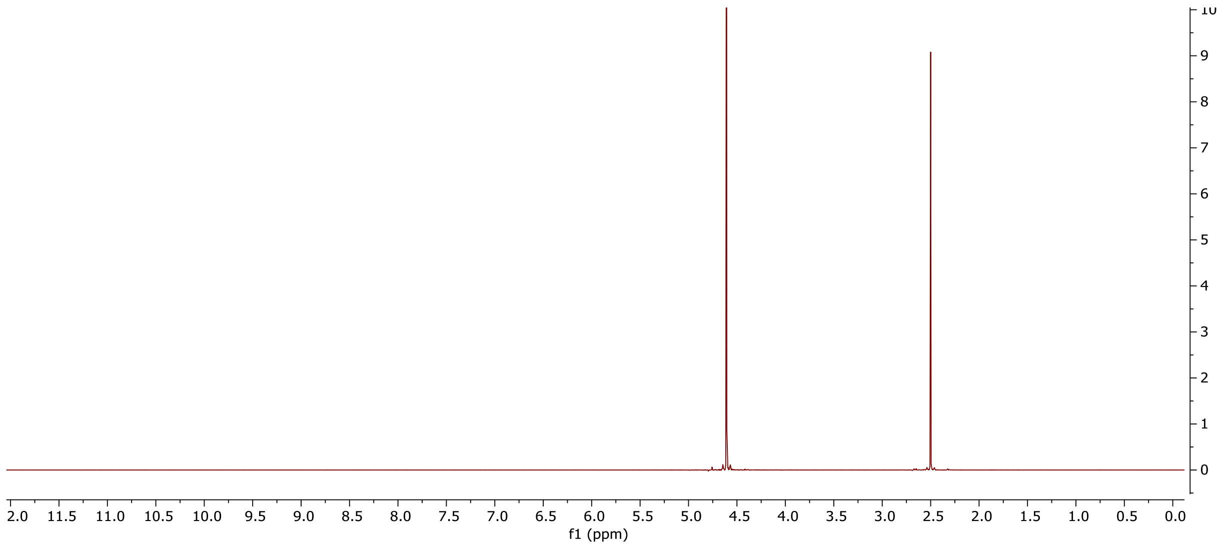

Figure S2. ^{1}H NMR of a portion (c.a. 1 cm) of DBS-CONHNH$_2$/alginate gel tube prepared in D_2O using 0.3% wt/vol of DBS-CONHNH$_2$ and 0.5% wt/vol of alginate.

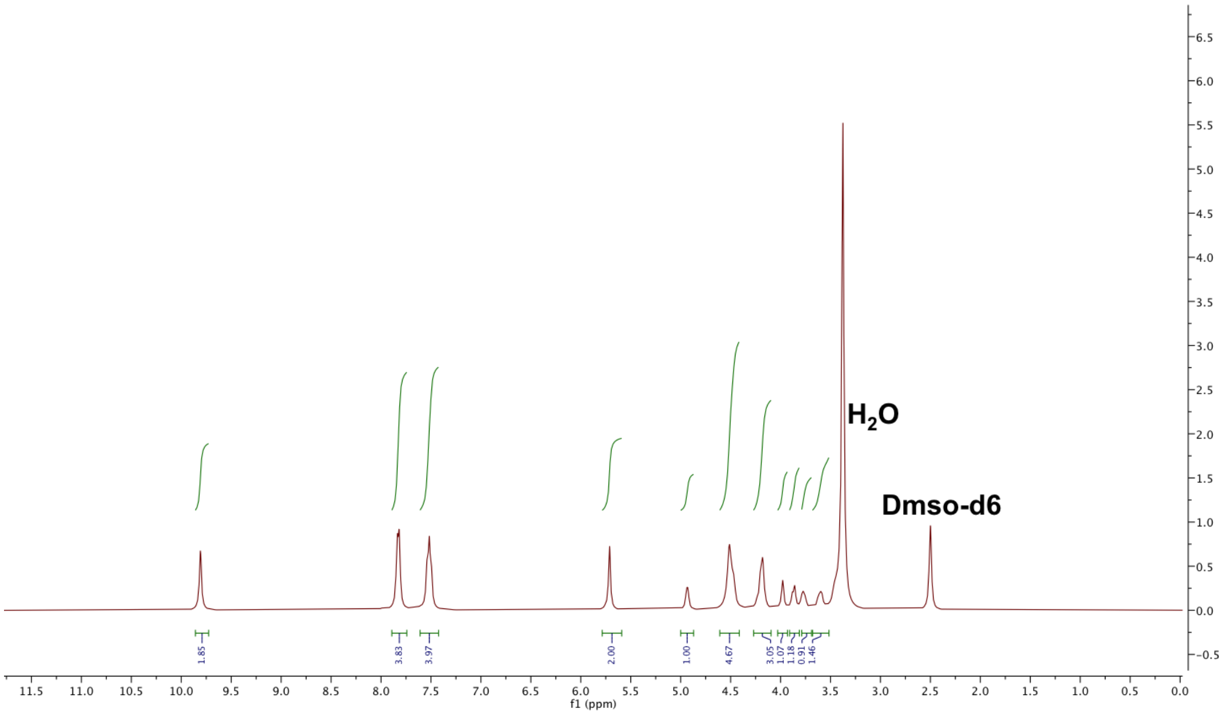

Figure S3. ^{1}H NMR of DBS-CONHNH$_2$ in DMSO-d6.

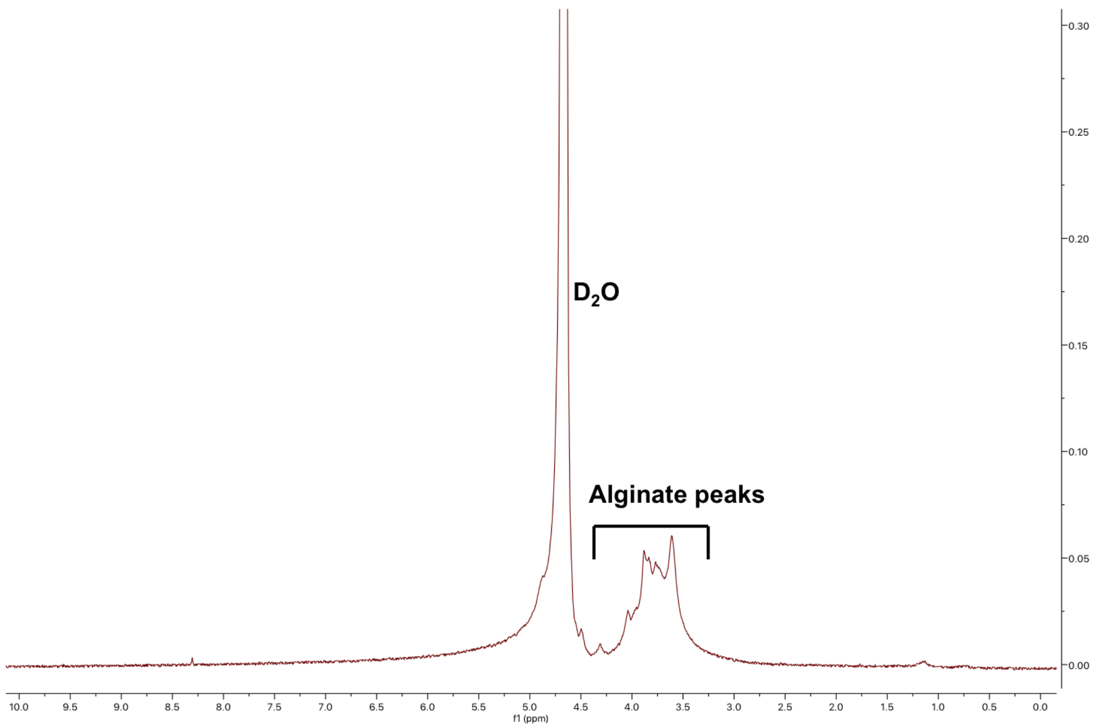

Figure S4. ^{1}H NMR of alginate in D_2O.

S2.2.2 NMR quantification of DBS-CONHNH$_2$ incorporated *into a DBS-CONHNH$_2$/alginate gel tube.* ^{1}H NMR was employed to quantify the exact amount of DBS-CONHNH$_2$ incorporated into DBS- CONHNH$_2$/alginate core-shell gel tubes. The gel tube used for this experiment was prepared by combining DBS-CONHNH$_2$ (0.3 % wt/vol) and alginate (0.5 % wt/vol) as described in Section S2.1. The obtained tube was isolated and dried under high vacuum. The resulting solid was dissolved in DMSO-d6 (0.7 mL), and acetonitrile (1.4 μL) was added as an internal standard. To make sure that all the DBS-CONHNH$_2$ was dissolved, the sample was ground and then sonicated for 30 min. The ^{1}H NMR spectrum was recorded and the concentration of the LMWG calculated by comparison of the integrals of relevant peaks (DBS-CONHNH$_2$ aromatic peaks δ = 7.53 and 7.83 ppm) to that of acetonitrile (δ = 2.09 ppm). To ensure the results were reproducible, this experiment was performed on two different batches of gel filaments. It is noted that due to the low solubility of alginate in DMSO- d6, the alginate peaks were not visible.

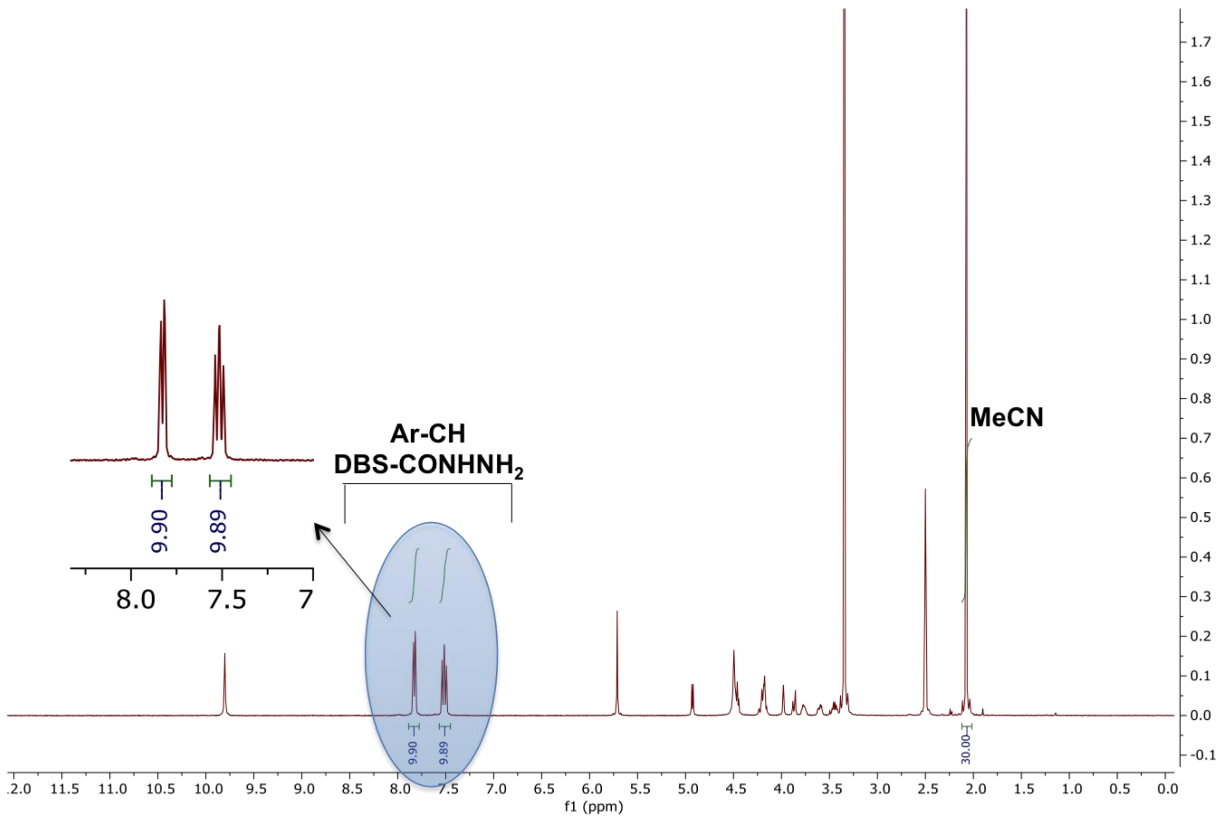

Figure S5. ^{1}H NMR of DBS-CONHNH$_2$ incorporated into a DBS-CONHNH$_2$/alginate gel tube prepared using 0.3% wt/vol DBS-CONHNH$_2$ and 0.5% wt/vol alginate.

S2.3 Infrared (IR) spectroscopy

DBS-CONHNH$_2$/alginate and alginate gel tubes were prepared as described in Section S2.1.. DBS-CONHNH$_2$ gels were prepared in sample vials as described in Section S4.1.. Xerogel samples for infrared were obtained by removing the solvent from the gels under high vacuum. A small amount of the resulting powder was placed into the infrared spectrophotometer and the spectra recorded in the range of 450-4000 cm^{-1}.

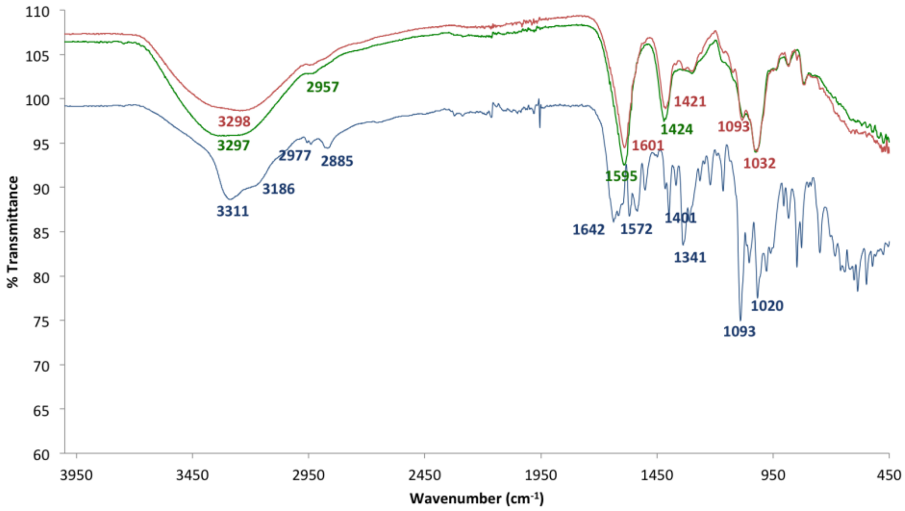

Figure S6. IR spectra of xerogels obtained from DBS-CONHNH$_2$ gel (0.3% wt/vol, blue line), alginate gel tube (0.8% wt/vol, green line) and DBS-CONHNH$_2$/alginate gel tube (containing 0.3% wt/vol of DBS-CONHNH$_2$ and 0.5% wt/vol alginate; red line).

S2.4 Optical microscopy

Optical microscopy images were collected on a Zeiss stereo microscope. The samples were dehydrated through an ethanol series, then embedded in LR white resin. Sections were 1 μm thick. Once the section was dried on the slide, it was stained with Toluidine Blue (0.6% with 0.3% Na_2CO_3).

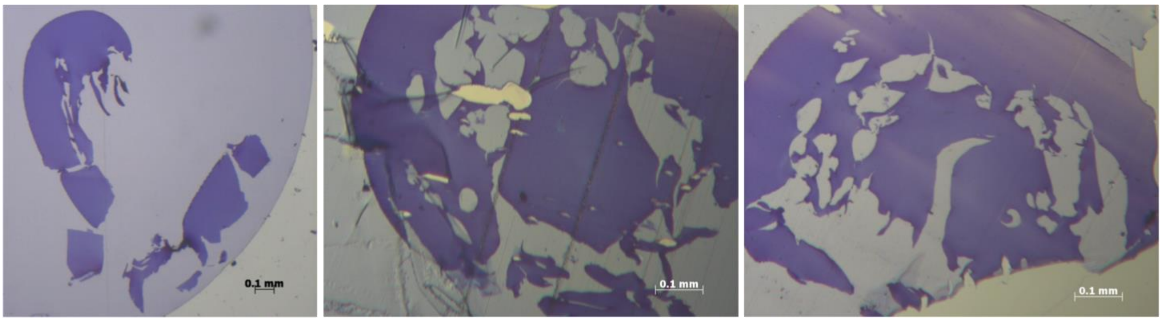

Figure S7. Fragmented cross-section of an alginate tube (0.8% wt/vol) embedded in resin and stained using toluidine blue (scale bars 0.1 mm).

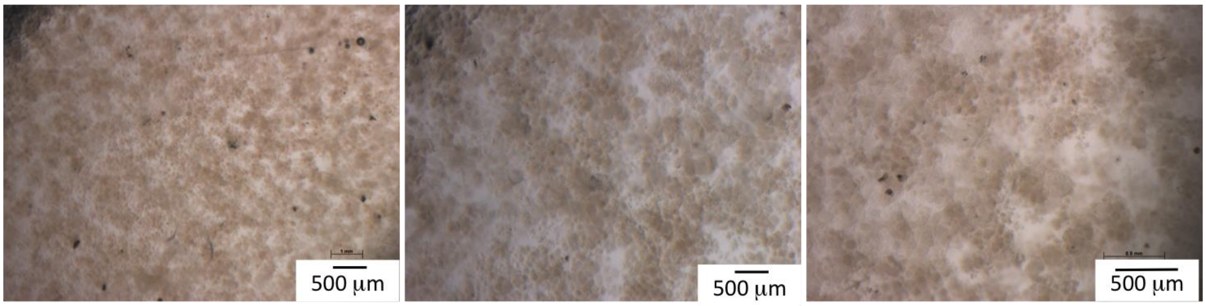

Figure S8. Optical microscopy images of DBS-$CONHNH_2$ gel prepared in a sample vial (0.3% wt/vol). Scale bars: 0.5 mm.

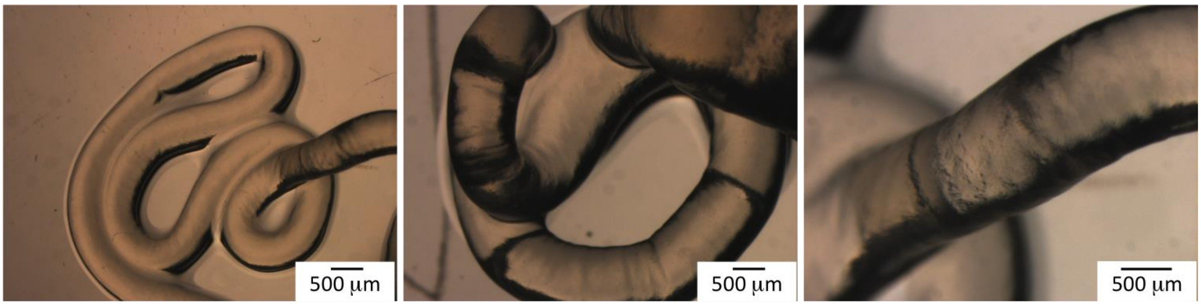

Figure S9. Optical microscopy images of alginate gel tube (0.8% wt/vol). Scale bars: 0.5 mm.

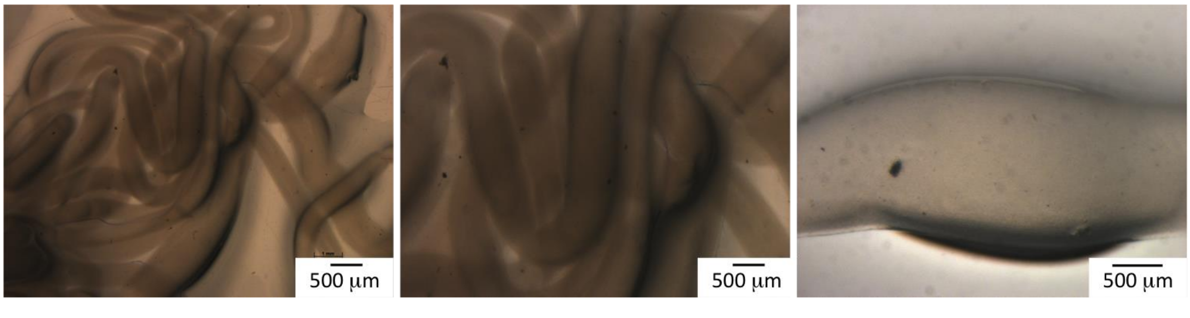

Figure S10. Optical microscopy images of DBS-$CONHNH_2$/alginate gel tube (0.3% wt/vol DBS-$CONHNH_2$ and 0.5% wt/vol alginate). Scale bars: 0.5 mm.

S2.5 Scanning Electron Microscopy (SEM)

S2.5.1 Preparation of samples for SEM. Samples for SEM were critical point dried (acetone and liquid CO_2) and mounted on stubs either as a portion, or halved using a razor blade. Mounted samples were sputter coated with Au/Pd.

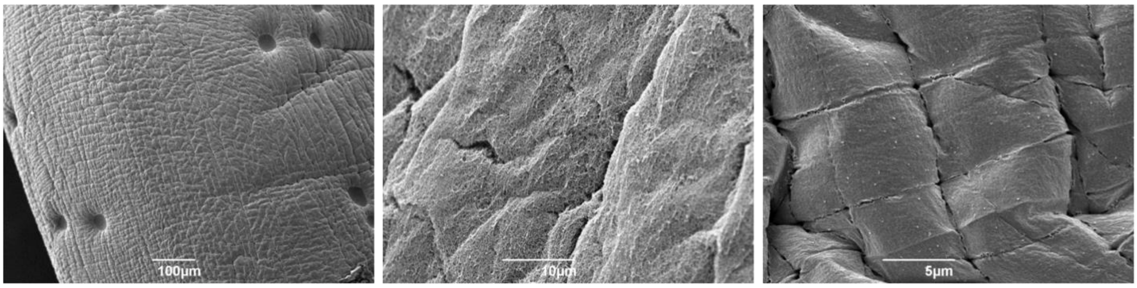

Figure S11. SEM images of DBS-$CONHNH_2$/alginate core-shell gel tube surface. Scale bars from left to right: 100, 10 and 5 µm.

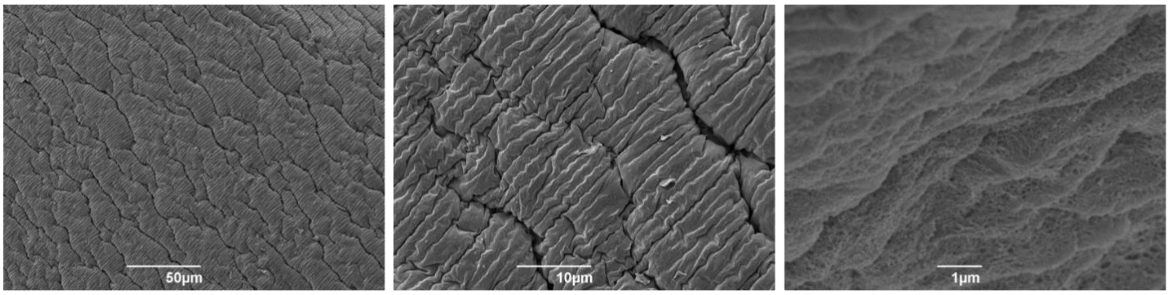

Figure S12. SEM images of alginate gel tube surface. Scale bars from left to right: 50, 10 and 1 µm.

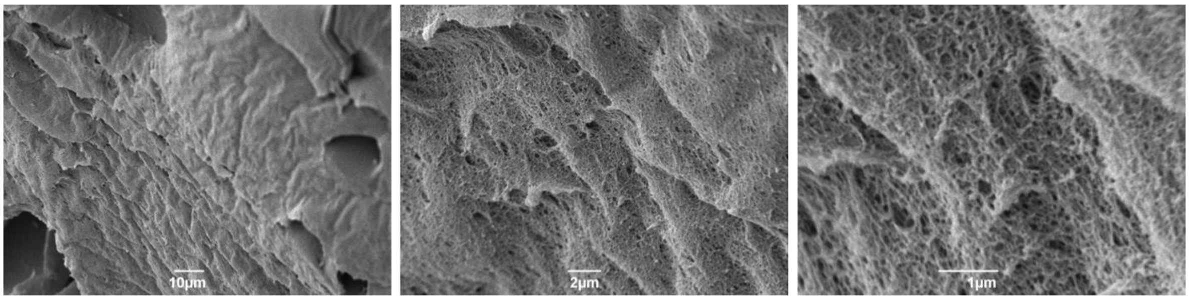

Figure S13. SEM images of alginate gel tube cross-section. Scale bars from left to right: 10, 2 and 1 µm.

S3 Preparation and characterisation of DBS-$CONHNH_2$ gel filaments by wet spinning

S3.1 Preparation of DBS-$CONHNH_2$ gel filaments

DBS-$CONHNH_2$ (1.5, 3.0 and 4.5% wt/vol) was dissolved in DMSO. The solution was transferred into a 1.0 mL volume syringe and injected through a blunt tip needle (30G, 23G, 20G, 18G or 15G) into a water bath (40 mL) using a syringe pump at a known flow rate (3.4, 6.8, 10.0, 20.0, 27.0 µL/min).

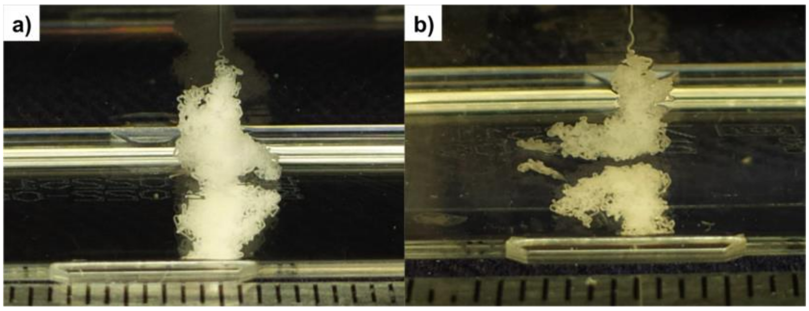

Figure S14. Photographic images of DBS-CONHNH$_2$ gel filament (4.5% wt/vol) prepared by wet spinning using a 23G (a) and a 20G (b) blunt tip needle at a 3.4 μL/min flow rate.

Table S1. Diameter of DBS-CONHNH$_2$ gel filaments by wet spinning under different conditions.

DBS-CONHNH$_2$ concentration	Flow rate (μL/min)	Needle inner diameter			
		30G	23G	20G	18G
		Gel filament average diameter			
1.5 % wt/vol	3.4	84 μm	93 μm	118 μm	-
	6.8	155 μm	-	-	-
3.0 % wt/vol	3.4	133 μm	122 μm	118 μm	90 μm
	6.8	114 μm	81 μm	101 μm	-
4.5 % wt/vol	3.4	152 μm	-	130 μm	-
	6.8	183 μm	-	130 μm	-

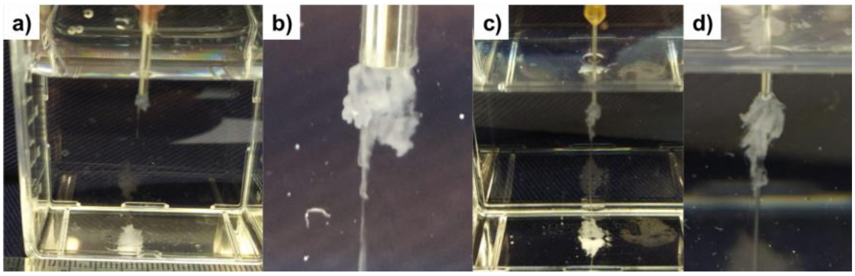

Figure S15. Photographic images of gel clog (a and b) and clog/filament transition (c and d) during the preparation of DBS-CONHNH$_2$ gel filaments (4.5% wt/vol) by wet spinning using a 15G blunt tip needle (a and b) and a 23G (c and d) blunt tip needle at a 3.4 μL/min flow rate.

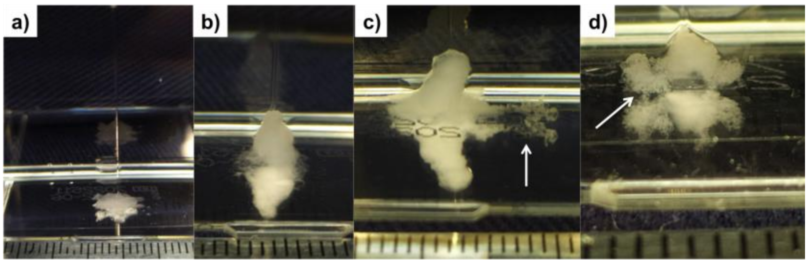

Figure S16. Photographic images of gelation at the bottom of the tank (a and b) and gelation/filament transition (c and d) during the preparation of DBS-CONHNH$_2$ gel filaments (4.5% wt/vol) by wet spinning using a 30G (a and b) and a 23G (c and d) blunt tip needle at a 20.0 μL/min flow rate. The white arrows show the partial formation of the gel filament.

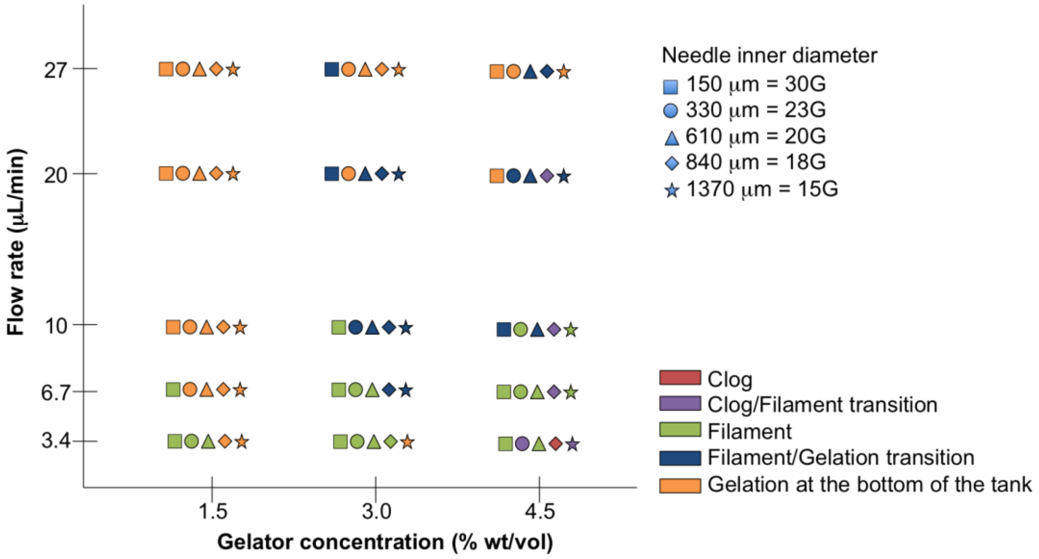

Figure S17. Flow rate/concentration phase diagrams for DBS-CONHNH$_2$ wet spinning.

S3.2 Optical Microscopy

DBS-CONHNH$_2$ gel filaments (3.0% wt/vol) for optical microscopy imaging were prepared by wet spinning as described in Section S3.1 using a 23G or a 30G blunt tip needle at a 3.4 μL/min flow rate.

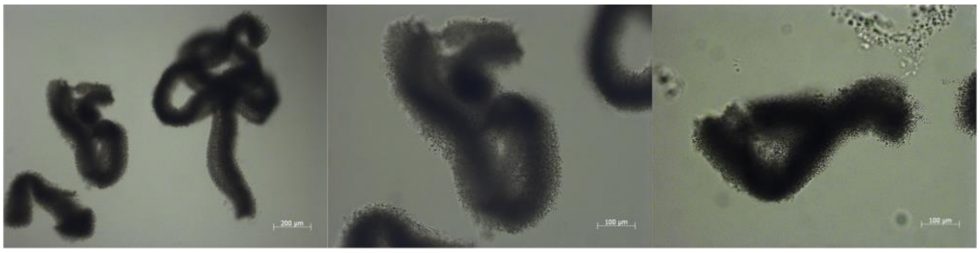

Figure S18. Bright-field optical microscopy images of DBS-CONHNH$_2$ gel filament portions (3.0% wt/vol) prepared by wet spinning using a 23G blunt tip needle at a 3.4 μL/min flow rate. Scale bars: 200 μm (left) and 100 μm (centre and right).

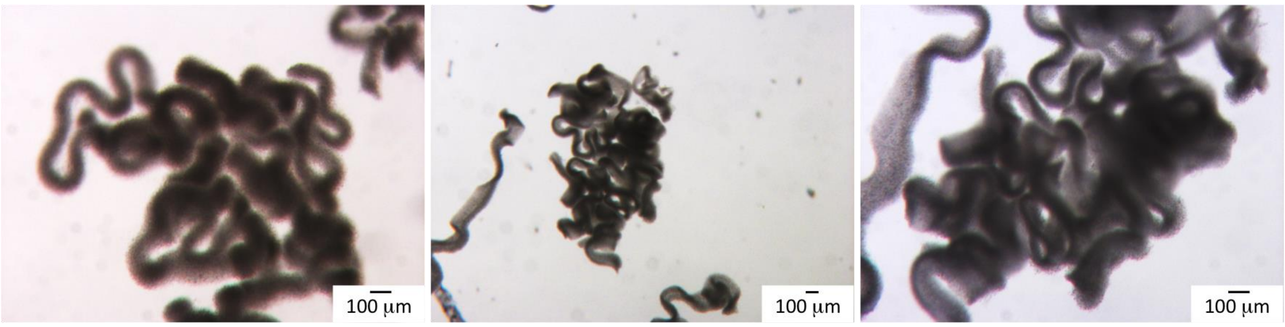

Figure S19. Optical microscopy images of DBS-CONHNH$_2$ gel filament portions (3.0% wt/vol; 23G blunt tip needle, 3.4 μL/min flow rate) freshly prepared (left) and after 30 days (centre and right). Scale bars: 100 μm.

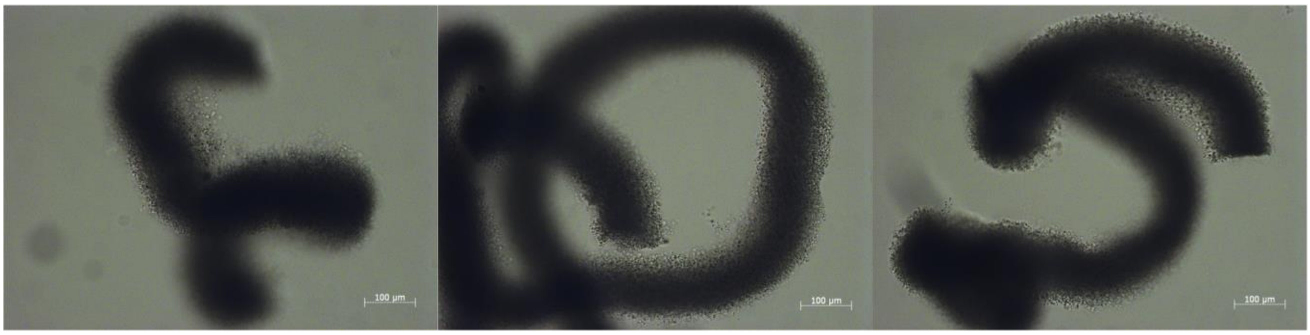

Figure S20. Bright-field optical microscopy images of DBS-CONHNH$_2$ gel filament portions (3.0% wt/vol) prepared by wet spinning using a 30G blunt tip needle at a 3.4 μL/min flow rate. Scale bars: 100 μm.

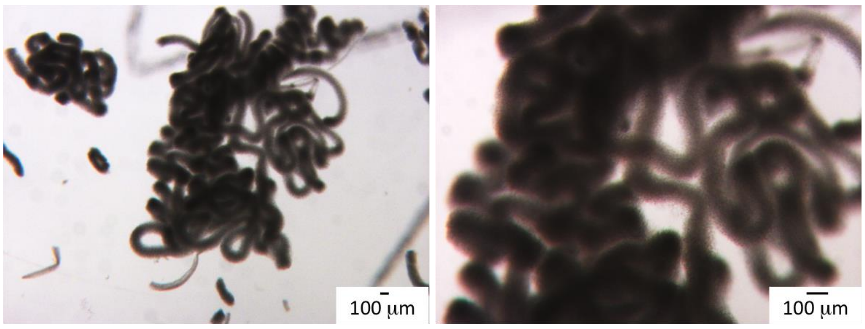

Figure S21. Optical microscopy images of freshly prepared DBS-CONHNH$_2$ gel filament portions (3.0% wt/vol; 30G blunt tip needle, 3.4 μL/min flow rate). Scale bars: 100 μm.

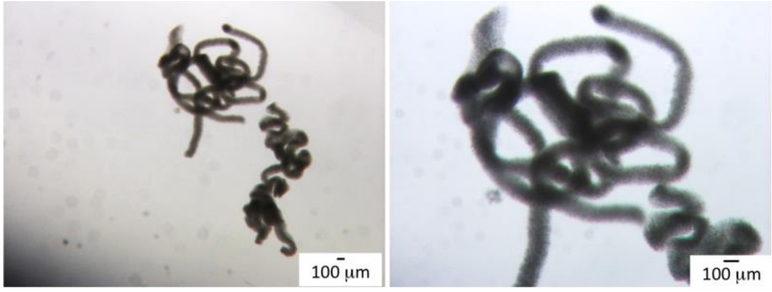

Figure S22. Optical microscopy images of DBS-CONHNH$_2$ gel filament portions (3.0% wt/vol; 30G blunt tip needle, 3.4 μL/min flow rate) after 30 days. Scale bars: 100 μm.

S3.3 Transmission and Scanning Electron Microscopy (TEM and SEM)

DBS-CONHNH$_2$ gel filaments (3.0% wt/vol) for TEM and SEM imaging were prepared by wet spinning as described in Section S3.1 using a 23G or a 30G blunt tip needle at a 3.4 μL/min flow rate. Sample preparation for SEM was carried out by freeze drying, whereas samples for TEM were obtained by placing a small amount of each sample on a copper grid. The excess of sample was removed with filter paper and allowed to set for 5 min. A negative stain (1% uranyl acetate) was then added and the samples were left to rest for 30 min before taking the images.

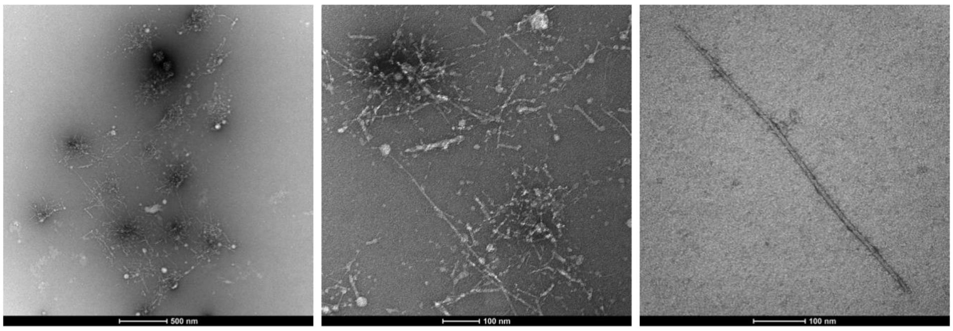

Figure S23. TEM images of DBS-CONHNH$_2$ gel filament (3.0% wt/vol; 30G blunt tip needle, 3.4 μL/min flow rate). Scale bars: 500 nm (left) and 100 nm (centre and right).

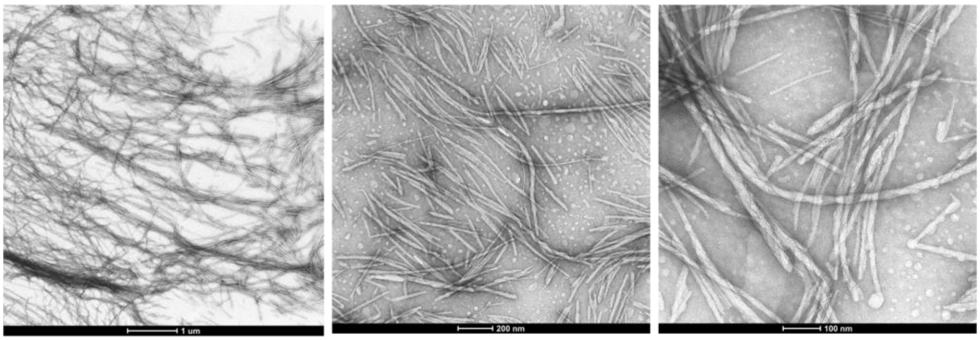

Figure S24. TEM images of DBS-CONHNH$_2$ bulk gel prepared in sample vials (0.3% wt/vol). Scale bars: 1μm (left), 200 nm (centre) and 100 nm (right).

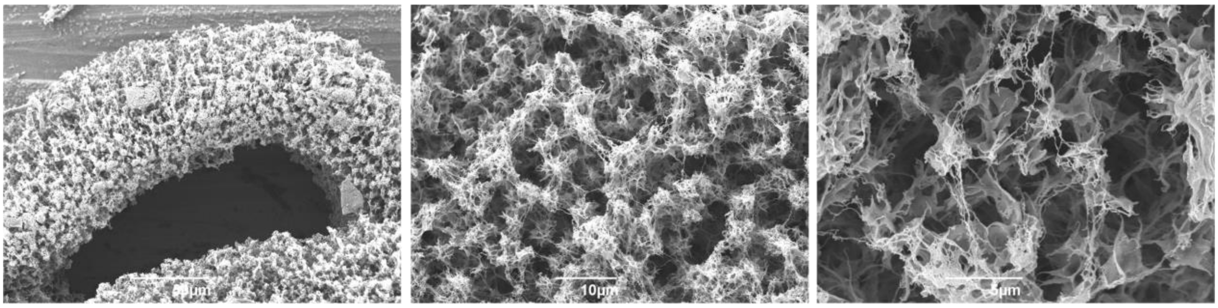

Figure S25. SEM images of DBS-CONHNH$_2$ gel filament (3.0% wt/vol; 23G blunt tip needle, 3.4 μL/min flow rate). Scale bars: 50 μm (left), 10 μm (centre) and 5 μm (right).

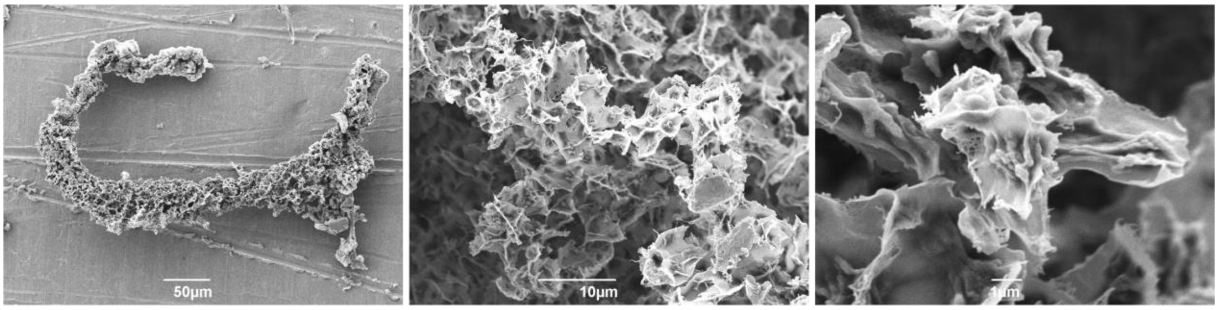

Figure S26. SEM images of DBS-CONHNH$_2$ gel filament (3.0% wt/vol; 30G blunt tip needle, 3.4 μL/min flow rate). Scale bars: 50 μm (left), 10 μm (centre) and 1 μm (right).

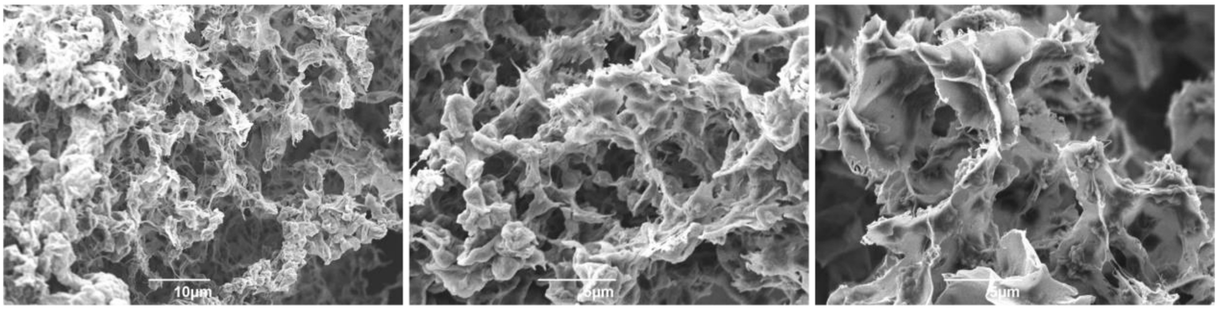

Figure S27. Additional SEM images of DBS-CONHNH$_2$ gel filament (3.0% wt/vol; 30G blunt tip needle, 3.4 μL/min flow rate). Scale bars: 10 μm (left) and 5 μm (centre and right).

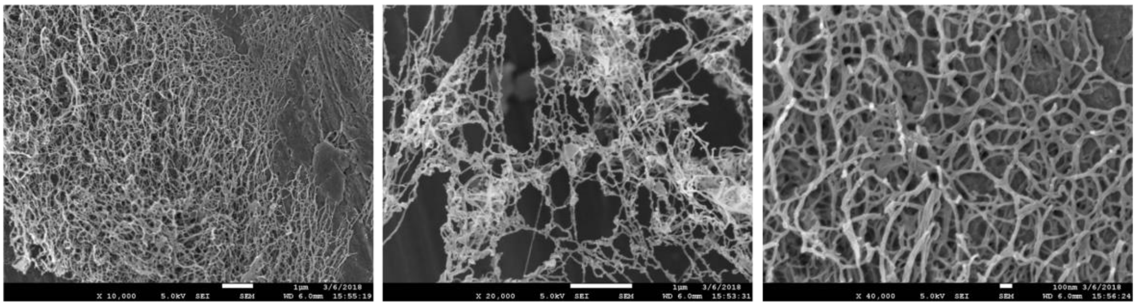

Figure S28. SEM images of DBS-CONHNH$_2$ bulk gel prepared in sample vials (0.3% wt/vol). Scale bars: 1μm (left and centre) and 100 nm (right).

S3.4 NMR assays

^{1}H NMR was used to quantify the amount of residual DMSO into the gel filaments prepared by wet spinning. The gel filaments used for this experiment was prepared by dissolving DBS-CONHNH$_2$ (1.5, 3.0 and 4.5 % wt/vol) in DMSO and injecting 50 μL of the solution into a water bath with a syringe pump at a 3.4 μL/min flow rate (23G blunt tip needle). The obtained filament was transferred to a vial and gently washed with D_2O twice. The solid was then transferred to a NMR tube containing D2O (0.75 mL) and acetonitrile (2.5 μL), which was added as an internal standard. The ^{1}H NMR spectrum was recorded and the concentration of residual DMSO was calculated by comparison of the integrals of relevant peaks (DMSO δ = 2.50 ppm) to that of acetonitrile (δ = 2.09 ppm).

The calculations were performed as follows. The integral of the DMSO peak was divided by the corresponding number of -CH (6 for DMSO) and then further divided by 10 (since MeCN was integrated as 30 instead of 3, to reduce the error in calculations). The result was multiplied by the μmol of MeCN added (*i.e.* 47.5 μmol) to give the μmol of residual DMSO, which were then converted into the corresponding volume.

Table S2 Amount of residual DMSO in filaments produced at different concentrations of DBS-CONHNH$_2$ after gentle washing two times in D_2O.

DBS-CONHNH$_2$ concentration in filament (50 μL)	MeCN signal integral (multiplied by 10)	DMSO signal integral	Amount of residual DMSO calculated (μmol)	Amount of residual DMSO calculated (μL)
1.5% wt/vol	30	0.27	0.21 μmol	0.015 μL
3.0% wt/vol	30	5.86	4.68 μmol	0.33 μL
4.5% wt/vol	30	9.82	7.84 μmol	0.55 μL

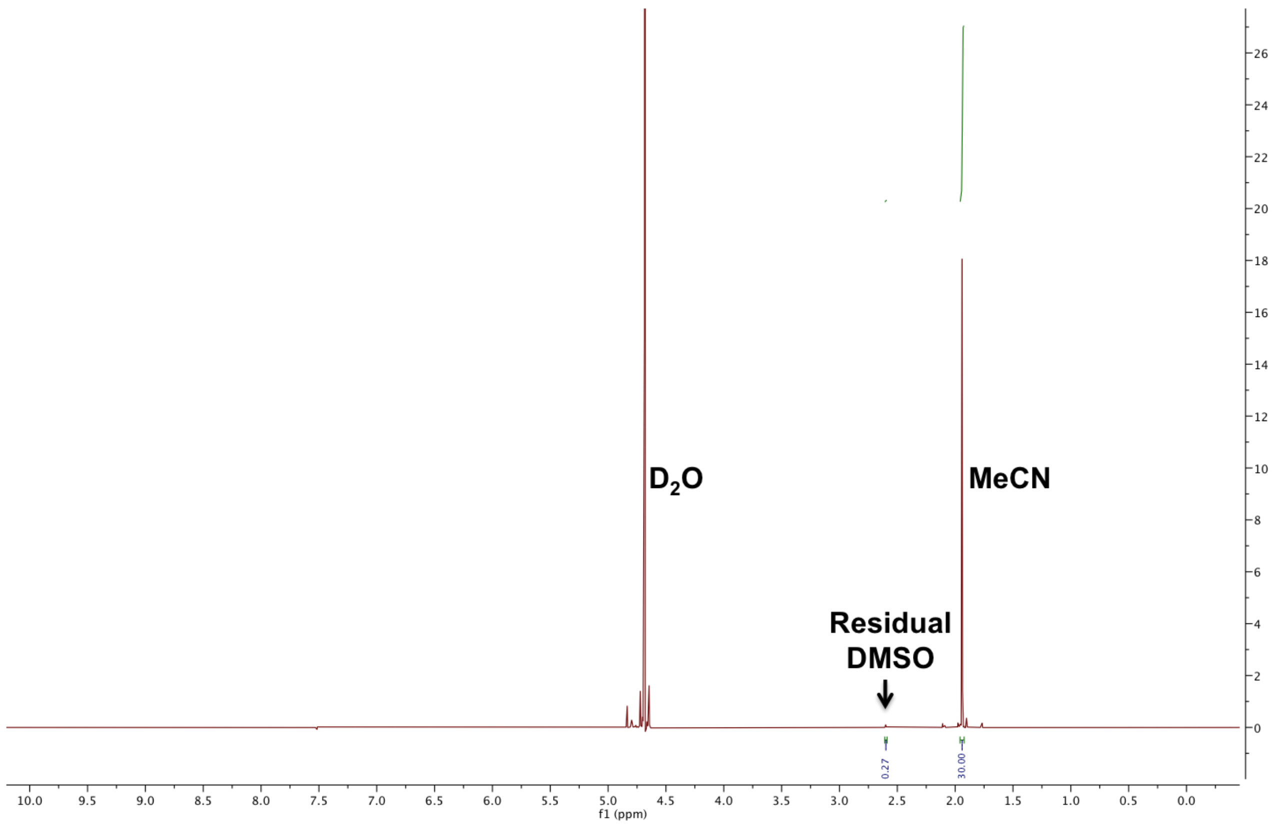

Figure S29. ^{1}H NMR of DBS-CONHNH$_2$ gel filament prepared by wet spinning (50 μL – 1.5% wt/vol; 23G blunt tip needle, 3.4 μL/min flow rate).

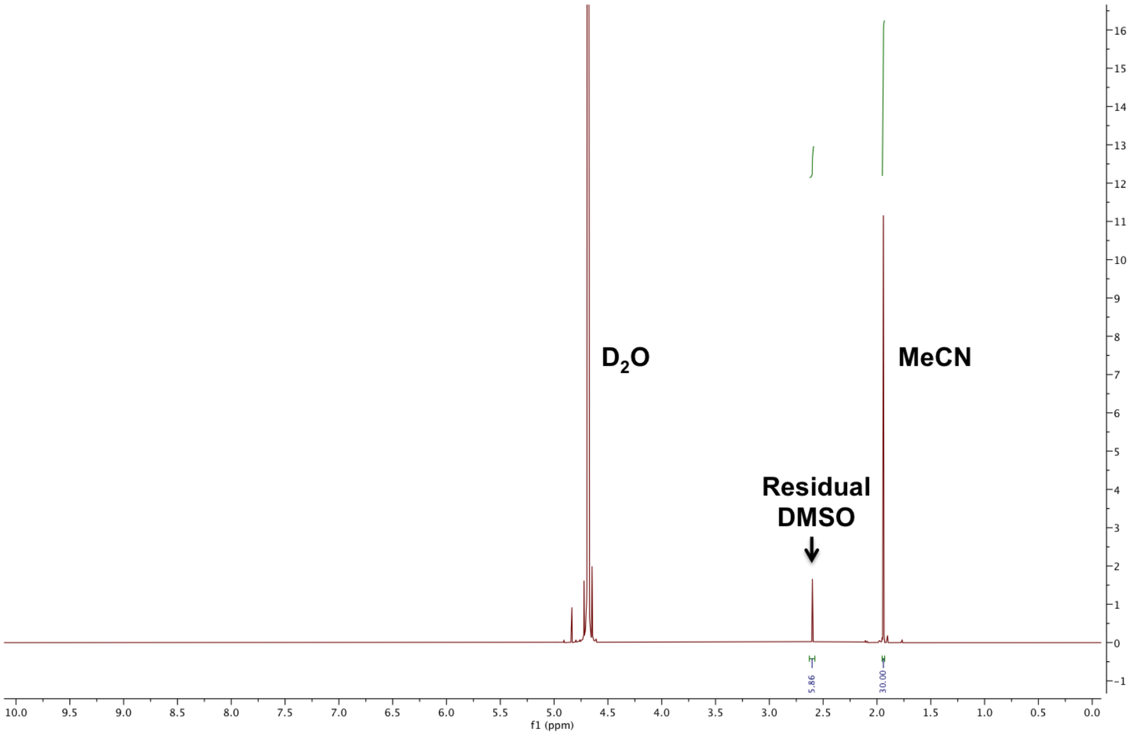

Figure S30. ^{1}H NMR of DBS-CONHNH$_2$ gel filament prepared by wet spinning (50 μL – 3.0% wt/vol; 23G blunt tip needle, 3.4 μL/min flow rate).

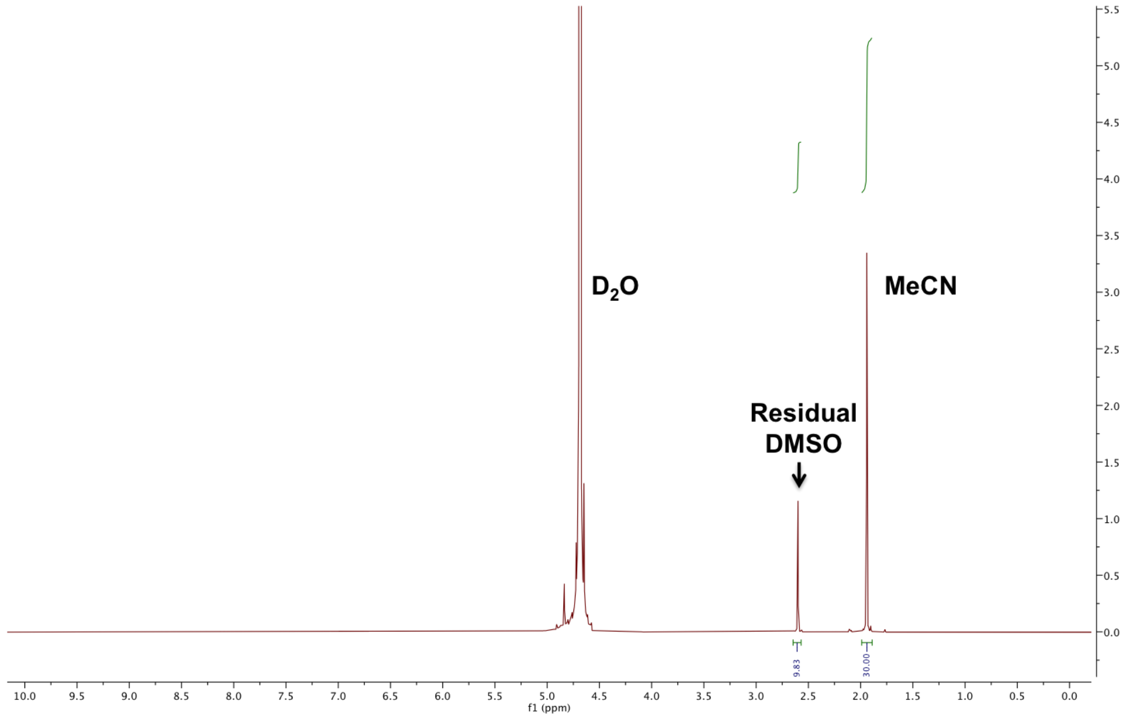

Figure S31. ^{1}H NMR of DBS-CONHNH$_2$ gel filament prepared by wet spinning (50 μL – 4.5% wt/vol; 23G blunt tip needle, 3.4 μL/min flow rate).

^{1}H NMR was also employed to quantify the exact amount of DBS-CONHNH$_2$ incorporated into the gel filaments prepared by wet spinning. The gel filaments used for this experiment was prepared by dissolving DBS-CONHNH$_2$ (1.5, 3.0 and 4.5 % wt/vol) in DMSO and injecting 50 μL of the solution into a water bath with a syringe pump at a 3.4 μL/min flow rate (23G blunt tip needle). The obtained filament was isolated and dried under high vacuum. The resulting solid was dissolved in DMSO-d6 (0.7 mL), and acetonitrile (2.0 μL) was added as an internal standard. To make sure that all the DBS-CONHNH$_2$ was dissolved, the sample was ground and then sonicated for 30 min. The ^{1}H NMR spectrum was recorded and the concentration of the LMWG calculated by comparison of the integrals of relevant peaks (DBS-CONHNH$_2$ aromatic peaks δ = 7.53 and 7.83 ppm) to that of acetonitrile (δ = 2.09 ppm).

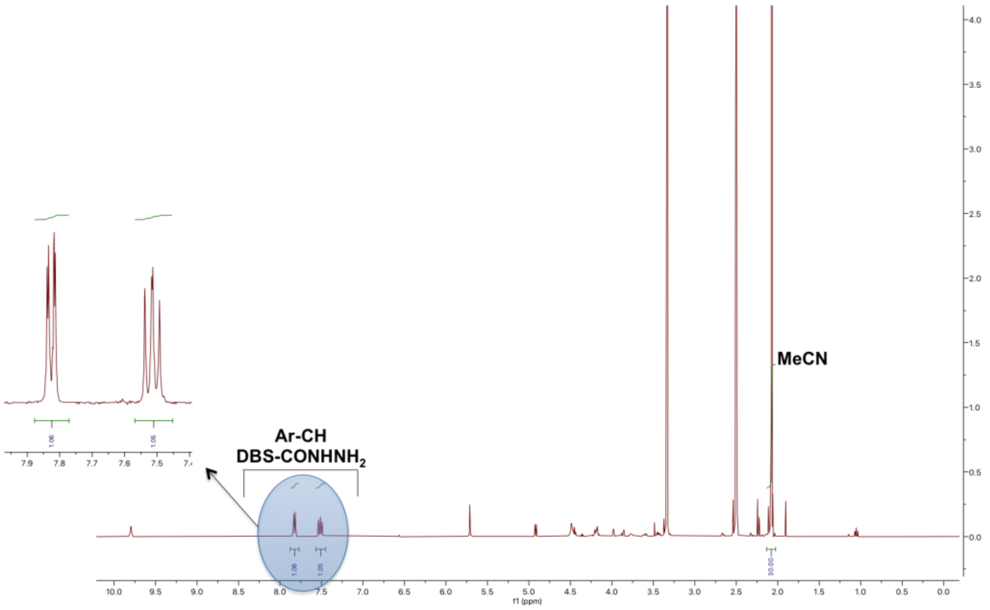

Figure S32. ^{1}H NMR of dried DBS-CONHNH$_2$ gel filament prepared by wet spinning (50 μL – 1.5% wt/vol; 23G blunt tip needle, 3.4 μL/min flow rate).

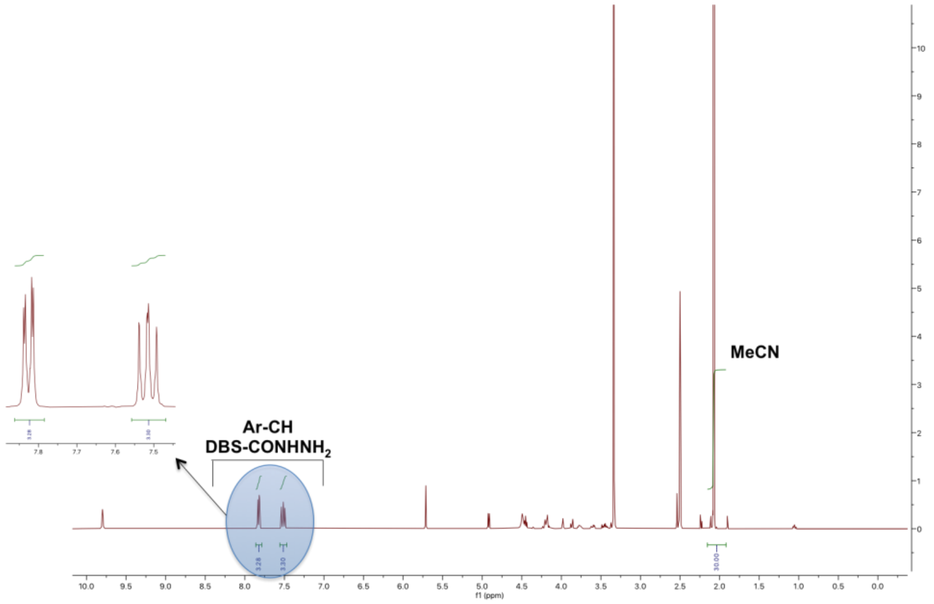

Figure S33. ^{1}H NMR of dried DBS-CONHNH$_2$ gel filament prepared by wet spinning (50 μL – 3.0% wt/vol; 23G blunt tip needle, 3.4 μL/min flow rate).

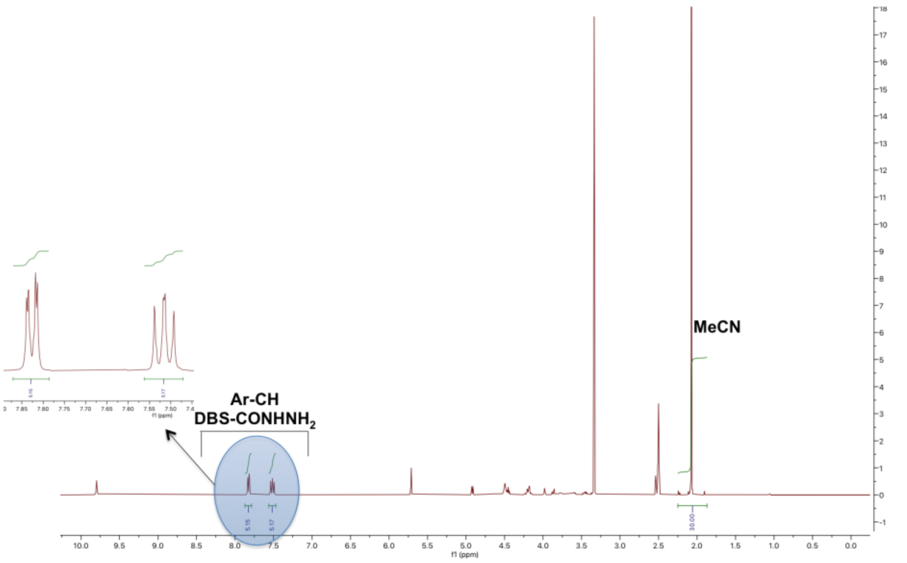

Figure S34. ^{1}H NMR of dried DBS-CONHNH$_2$ gel filament prepared by wet spinning (50 μL – 4.5% wt/vol; 23G blunt tip needle, 3.4 μL/min flow rate).

S3.5 Infrared (IR) spectroscopy

Xerogel samples for infrared were prepared by removing the solvent from the gels under high vacuum. A small amount of the resulting powder was placed into the infrared spectrophotometer and the spectra recorded in the range of 450-4000 cm^{-1}.

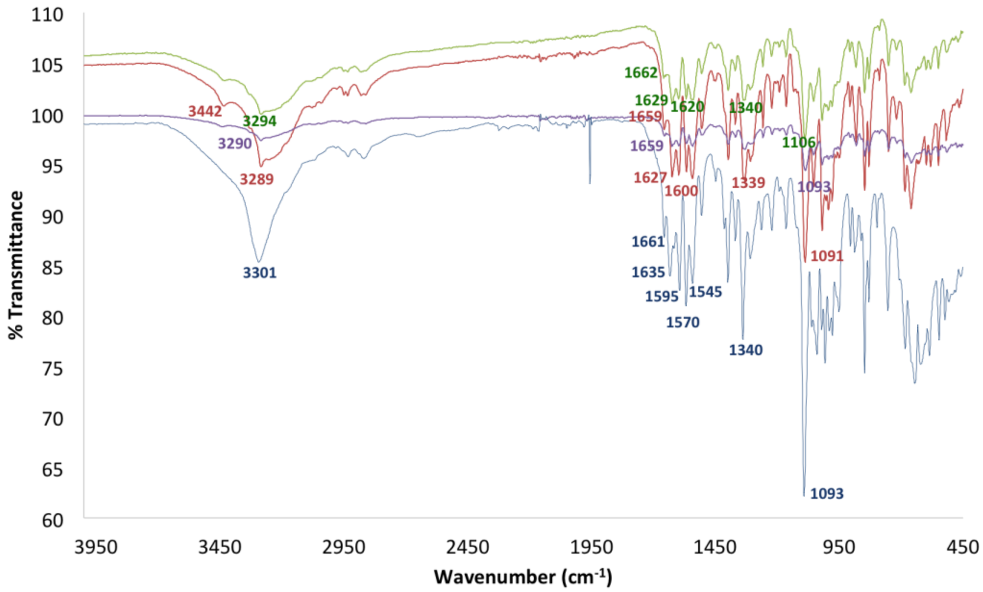

Figure S35. IR spectra of DBS-CONHNH$_2$ solid (blue line) and DBS-CONHNH$_2$ gel filaments: 1.5% wt/vol (purple line), 3.0% wt/vol (green line) and 4.5% wt/vol (red line).

S3.6 3D printing

For 3D-printing, a drawing /writing robot (Axidraw V3) was coupled with a syringe pump (Fusion 100CR from Chemyx Inc.). The Z-axis was moved manually by using a micrometric Z-moving platform. The pattern was drawn with Inkscape software. Multiple layers are made by stacking as many replicates of the initial pattern as desired. The print velocity of the Axidraw is set at 2% (4 mm/s), with a constant speed and low acceleration. Before printing, the distance between the tip and the drawing surface is set at 300 µm, by stacking three 100 µm thick cover slides and bringing the tip in contact with the cover slides. Then the cover slides are then removed. Photos and videos of the gels were captured with a DinoLite digital microscope (AM7515MZTL).

The drawing is made in 8 cm diameter polystyrene Petri dishes, filled with 25 mL of ultrapure water at room temperature. Solutions of gelators in dimethylsulfoxide are prepared at a concentration of 0.063 M for both gelators (corresponding to 30 mg/mL for DBS-CONHNH$_2$ (MW = 474.17) and 18.5 mg/mL for N-heptyl-D-galactonamide (GalC7) (MW = 293.3). The gelator solutions were extruded with a blunt-tip needles of 30G gauge (internal diameter 160 µm), at 5 µL/min in the water bath, with a distance tip-drawing surface of 300 µm. The Z-platform was moved up of 75 µm per layer for DBS-CONHNH$_2$ hydrogel and 50 µm for N-heptyl-D-galactonamide hydrogel.

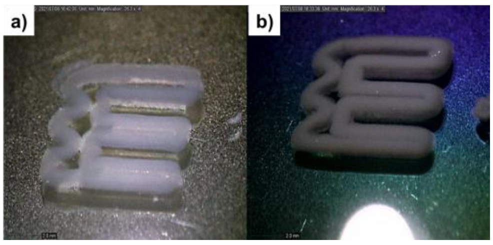

Figure S36. Photographic images of DBS-CONHNH$_2$ (a) and GalC7 (b) 3D printed gels by wet spinning (respectively 3.0 and 1.85% wt/vol) immediately after printing.

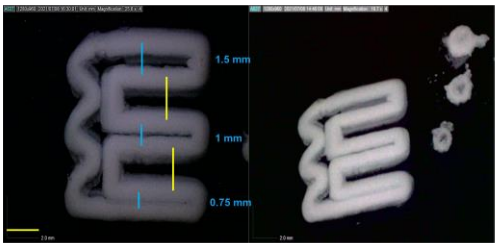

Figure S37. DinoLite microscopy images of GalC7 3D printed gels by wet spinning (1.85% wt/vol) immediately after printing.

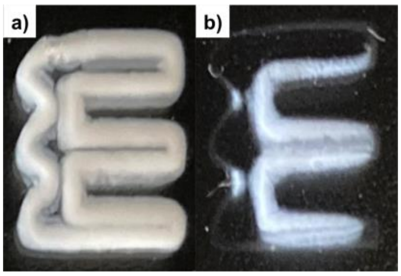

Figure S38. Photographic images of GalC7 3D printed gels by wet spinning (1.85% wt/vol) immediately after printing (a) and after 24h (b).

S4 Preparation and characterisation of DBS-CONHNH$_2$, DBS-CONHNH$_2$/alginate and alginate gels loaded with Au nanoparticles (NPs)

S4.1 Gel preparation

S4.1.1 DBS-CONHNH$_2$/alginate and alginate gel filaments. DBS-CONHNH$_2$/alginate and alginate gel filaments were prepared as described in Section S2.1.

S4.1.2 DBS-CONHNH$_2$/alginate gels in sample vials (for thermal stability and rheology studies). DBS-CONHNH$_2$ (0.3% wt/vol in 1 mL final total volume) was suspended in water (0.5 mL) and sonicated to help the dispersion of the solid particles. An aqueous alginate solution (1.0% wt/vol - 0.5 mL) was then added. The resulting suspension was heated until complete dissolution of the DBS-CONHNH$_2$. The sample was left undisturbed for few hours to allow the formation of the DBS-CONHNH$_2$ network. A solution of $CaCl_2$ (5.0 % wt/vol – 1 mL) was then added on top of each gel to crosslink the alginate chains for 30 min. The excess of $CaCl_2$ solution was then removed and the gels were washed with water multiple times.

S4.1.3 Alginate gels in sample vials (for thermal stability and rheology studies). Alginate gels were prepared by adding a $CaCl_2$ solution (5.0% wt/vol, 1 mL) to an aqueous alginate solution (0.8% wt/vol, 1 mL). Gelation occurred immediately. The excess of $CaCl_2$ solution was then removed and the gels were washed with water multiple times.

S4.1.4 DBS-CONHNH$_2$ gel filaments by wet spinning. DBS-CONHNH$_2$ gel filaments were prepared by wet spinnning (1.5, 3.0 and 4.5% wt/vol) at a flow rate of 3.4 μL/min, using a 23G blunt tip needle, as described in Section S3.1.

S4.1.5 DBS-CONHNH$_2$ gels in sample vials. DBS-CONHNH$_2$ (0.3 or 0.4% wt/vol) was suspended in water (1 mL). The suspension was sonicated to help the dispersion of the solid particles and then heated until

complete dissolution of the compound. The sample was left undisturbed to cool, allowing gel formation in few minutes.

S4.2 *In situ* formation of AuNPs

To induce the *in situ* formation of Au nanoparticles into gels, each gel was thoroughly washed with water multiple times and immersed in 1 mL of $AuCl_3$ solution (5, 10 or 20 mM) for 24 hours. After 24 hours, the supernatant was gently removed with a pipette and the gels were washed with water multiple times. A colour change was observed in the samples in which the Au was reduced from Au (III) to Au (0).

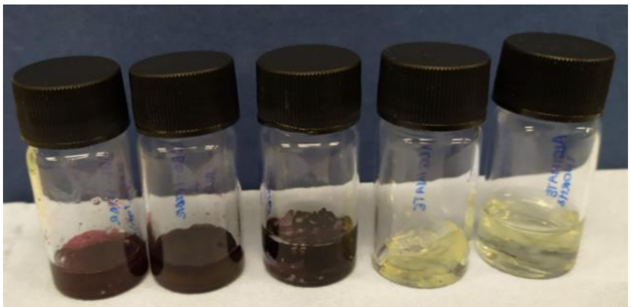

Figure S39. Photographic images of gels loaded with AuNPs. From left to right: DBS-$CONHNH_2$ gel in vial (0.3% wt/vol), DBS-$CONHNH_2$/alginate gel in vial (0.3% wt/vol DBS-$CONHNH_2$ and 0.5% wt/vol alginate), DBS-$CONHNH_2$/alginate core-shell gel filament (0.3% wt/vol DBS-$CONHNH_2$ and 0.5% wt/vol alginate), alginate gel in vial (0.8% wt/vol), alginate gel filament (0.8% wt/vol).

S4.2 Transmission Electron Microscopy (TEM)

Samples for TEM imaging were prepared as described in Sections S2.1 and S3.1, loaded with Au NPs (Section S4.2) and then treated for TEM analysis. A small amount of each sample was placed on a copper grid. The excess of sample was removed with filter paper and allowed to rest for 30 min before taking the images.

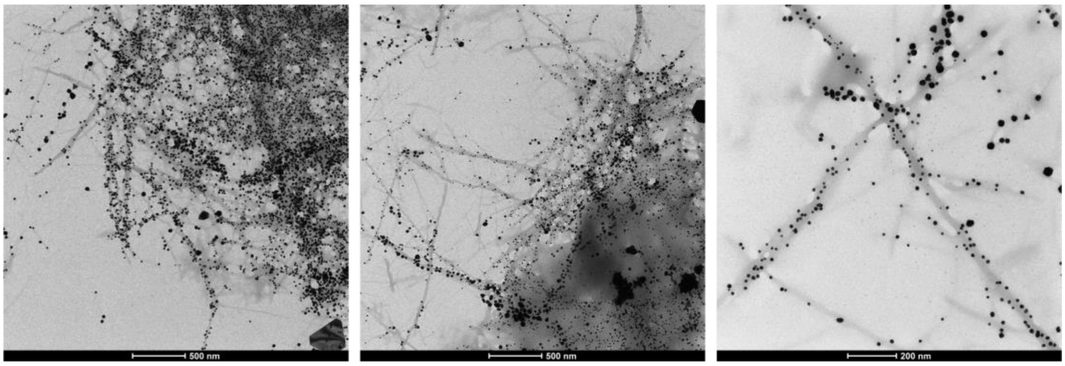

Figure S40. TEM images of DBS-$CONHNH_2$ bulk gel incorporating AuNPs (scale bars: 500 nm (left and centre) and 200 nm (right).

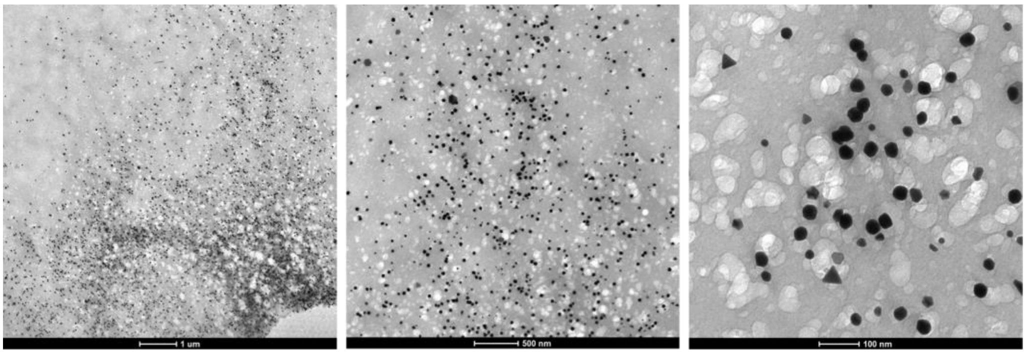

Figure S41. TEM images of DBS-CONHNH$_2$/alginate core-shell gel filaments incorporating AuNPs (scale bars from left to right: 1 µm, 500 nm and 100 nm).

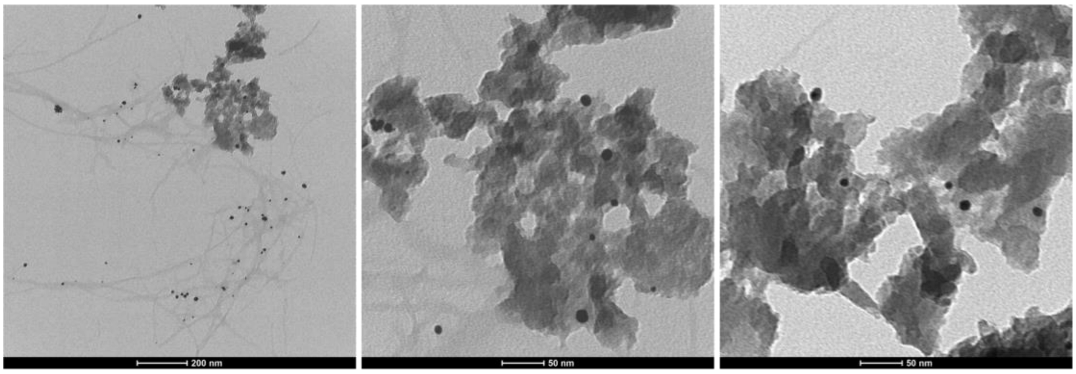

Figure S42. TEM images of DBS-CONHNH$_2$ wet spinning gel filament incorporating AuNPs (scale bars: 200 nm (left) and 50 nm (centre and right).

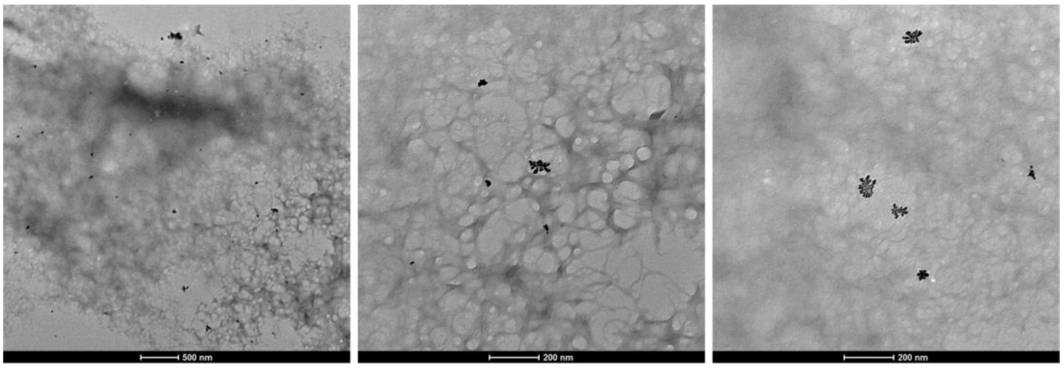

Figure S43. TEM images of alginate gel filament incorporating AuNPs. Scale bars: 500 nm (left) and 200 nm (centre and right).

S4.3 Uptake of Au(III)

S4.3.1 *Maximum uptake of Au(III) into DBS-CONHNH$_2$, alginate and DBS-CONHNH$_2$/alginate core-shell filaments and gels prepared in sample vials.* The gels used to estimate the uptake of Au (III) were prepared in water (1 mL) in sample vials or as gel filaments, as described in sections S2.1 and S4.1. Each of these gels was thoroughly washed with water multiple times and immersed in 1 mL of a 20mM $AuCl_3$ solution (containing 20.0 µmoles of Au(III)) for 24 hours. After 24 hours, an aliquot of the supernatant (50 µL) was removed, diluted to 2 mL and transferred to a cuvette. The UV absorbance at 305-310 nm was then measured giving the µmoles of Au(III) that was not incorporated into the gels. This was subtracted from the

initial μmoles of Au(III) added, to give the μmoles of Au(III) incorporated. To ensure data reproducibility, this experiment was performed in triplicate for each gel and average values are reported.

Table S3. Evaluation of Au(III) uptake into DBS-CONHNH$_2$, alginate and DBS-CONHNH$_2$/alginate hybrid gels by UV spectroscopy.

Gel	Loading of DBS-CONHNH$_2$ (wt/vol)	Loading of Alginate (wt/vol)	μmoles of Au (III) incorporated / mL of gel	% of Au (III) incorporated
DBS-CONHNH$_2$ gel in vial	0.3 %	-	16.5	82.8 %
Alginate gel filament	-	0.8 %	7.20	35.8 %
Alginate gel in vial	-	0.8 %	7.90	39.9 %
Hybrid gel filament	0.3 %	0.5 %	15.7	78.4 %
Hybrid gel in vial	0.3 %	0.5 %	14.7	73.7 %

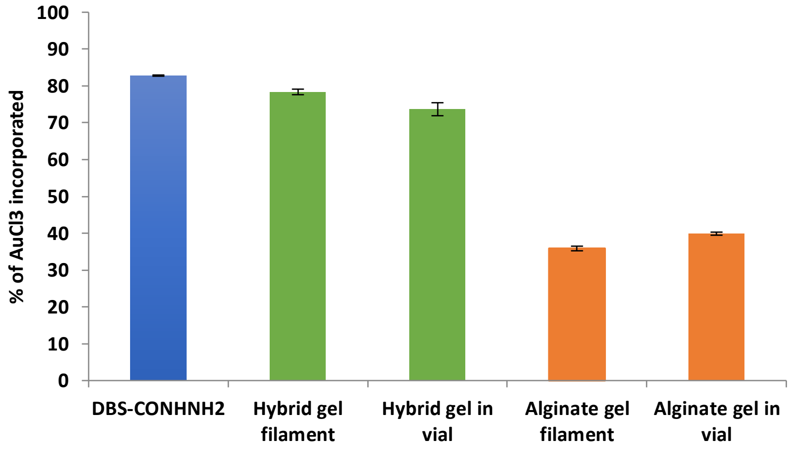

Figure S44. Percentage of Au(III) incorporated into DBS-CONHNH$_2$, alginate and DBS-CONHNH$_2$/alginate hybrid gels.

S4.3.2 *Uptake rate of Au(III) into DBS-CONHNH$_2$, alginate and DBS-CONHNH$_2$/alginate gels.* The gels used to estimate the uptake rate of Au(III) were prepared in water (1 mL) in sample vials or as gel filaments, as described in sections S2.1 and S4.1. Each of these gels was thoroughly washed with water multiple times and immersed in 2 mL of a 2.5 mM $AuCl_3$ solution (containing 5.0 μmoles of Au (III)). At regular time intervals (1, 3, 5 and 24 hours), an aliquot of the supernatant (150 μL) was removed, diluted to 2 mL and transferred to a cuvette. The UV absorbance at 305-310 nm was then measured. To ensure data reproducibility, this experiment was performed in triplicate for each gel and average values are reported.

Table S4. Evaluation of Au (III) uptake rate into DBS-CONHNH$_2$, alginate and DBS-CONHNH$_2$/alginate hybrid gels by UV spectroscopy.

Time (hours)	% of Au (III) incorporated				
	DBS-CONHNH$_2$ gel in vial (0.3% wt/vol)	Hybrid gel tube (0.3% wt/vol DBS-CONHNH$_2$ and 0.5% wt/vol alginate)	Hybrid gel in vial (0.3% wt/vol DBS-CONHNH$_2$ and 0.5% wt/vol alginate)	Alginate gel tube wt/vol)	Alginate gel in vial (0.8% wt/vol)
1h	38.33%	41.83%	24.71%	13.64%	20.72%
3h	75.53%	66.04 %	52.77%	19.70%	17.91%
5h	85.91%	88.45 %	87.49%	23.68%	15.77%
24h	100.0 %	100.0 %	100.0 %	22.37%	19.28%

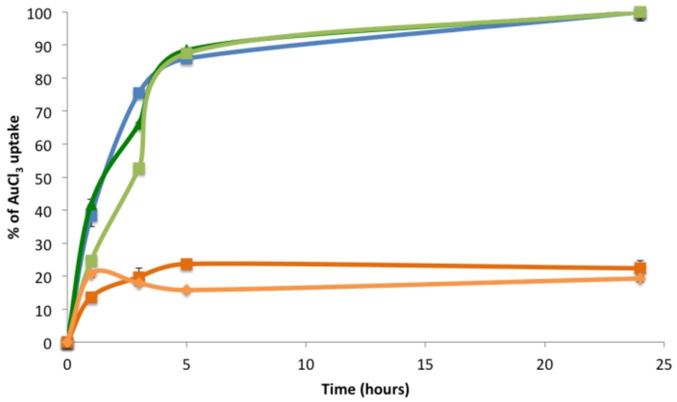

Figure S45. Percentage of Au(III) uptake over time into DBS-CONHNH$_2$ gel (prepared in a sample vial, 0.3% wt/vol – blue line), alginate gel (prepared in a sample vial, 0.8% wt/vol – light orange), alginate gel tube (0.8% wt/vol – dark orange), DBS-CONHNH$_2$/alginate hybrid gel (prepared in a sample vial, 0.3% wt/vol of DBS-CONHNH$_2$ and 0.8% wt/vol of alginate – light green) and DBS-CONHNH$_2$/alginate tube (0.3% wt/vol of DBS-CONHNH$_2$ and 0.8% wt/vol of alginate – dark green).

S4.3.3 *Uptake of Au (III) into DBS-CONHNH$_2$ gel filaments prepared by wet spinning.* The gel filaments used to estimate the uptake of Au(III) were prepared by wet spinnning (50 μL volume - 1.5, 3.0 and 4.5% wt/vol), as described in Section S4.1. After the filaments were formed, the water was gently removed and they were immersed in 1 mL of a 20 mM $AuCl_3$ solution (containing 20 μmoles of Au (III)) for 24 hours. After 24 hours, an aliquot of the supernatant (50 μL) was removed, diluted to 2 mL and transferred to a cuvette. The UV absorbance at 305-310 nm was then measured giving the μmoles of Au (III) that was not incorporated into the gels. This was subtracted from the initial μmoles of Au (III) added, to give the μmoles of Au (III) incorporated. To ensure data reproducibility, this experiment was performed in triplicate for each gel and average values are reported.

Table S5. Evaluation of Au(III) uptake by UV spectroscopy into DBS-CONHNH$_2$ gel filaments (50 μL - 1.5, 3.0 and 4.5% wt/vol) prepared by wet spinning.

Gel	Loading of DBS-CONHNH$_2$ (wt/vol)	Volume of gel	μmoles of Au (III) incorporated into gel	μmoles of Au (III) incorporated / mL of gel	% of Au (III) loaded incorporated
DBS-CONHNH$_2$ filament	1.5%	50 μL	6.35	127.0	27.0%
DBS-CONHNH$_2$ filament	3.0%	50 μL	9.53	190.6	40.5%
DBS-CONHNH$_2$ filament	4.5%	50 μL	14.93	298.6	63.5%

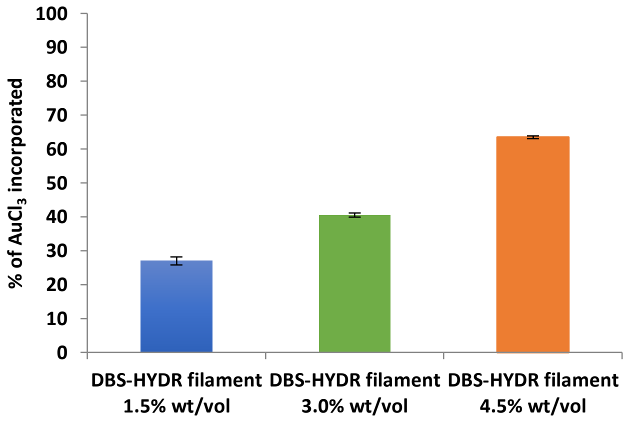

Figure S46. Percentage of Au(III) incorporated into DBS-CONHNH$_2$ gel filaments (50 μL - 1.5, 3.0 and 4.5% wt/vol) prepared by wet spinning.

S4.4 Infrared (IR) spectroscopy

Gels for IR were loaded with AuNPs by immersion in $AuCl_3$ (1mL, 5mM), as described in Section 4.2. Xerogel samples were prepared by removing the solvent from the gels under high vacuum. A small amount of the resulting powder was placed into the infrared spectrophotometer and the spectra recorded in the range of 450-4000 cm^{-1}.

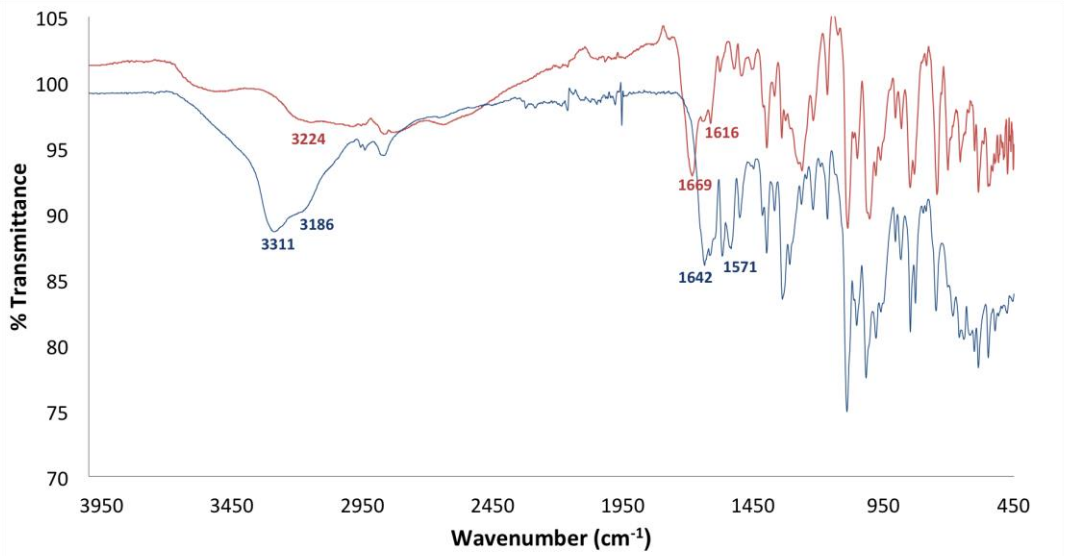

Fig. S47. IR spectra of DBS-CONHNH$_2$ bulk gel (0.3% wt/vol) with (red line) and without (blue line) AuNPs.

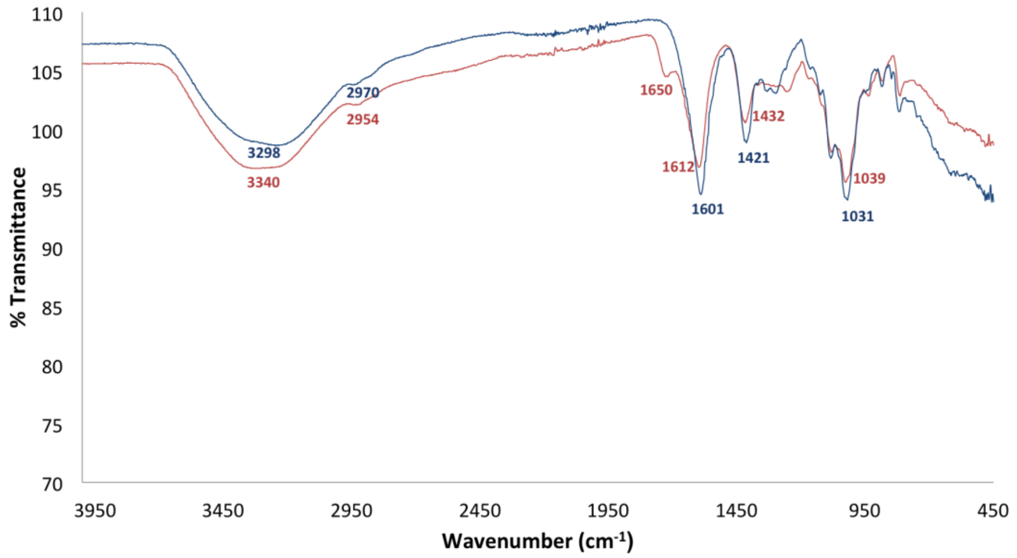

Figure S48. IR spectra of DBS-CONHNH$_2$/alginate core-shell gel filaments (0.3% wt/vol DBS-CONHNH$_2$ and 0.5% wt/vol alginate) with (red line) and without (blue line) AuNPs.

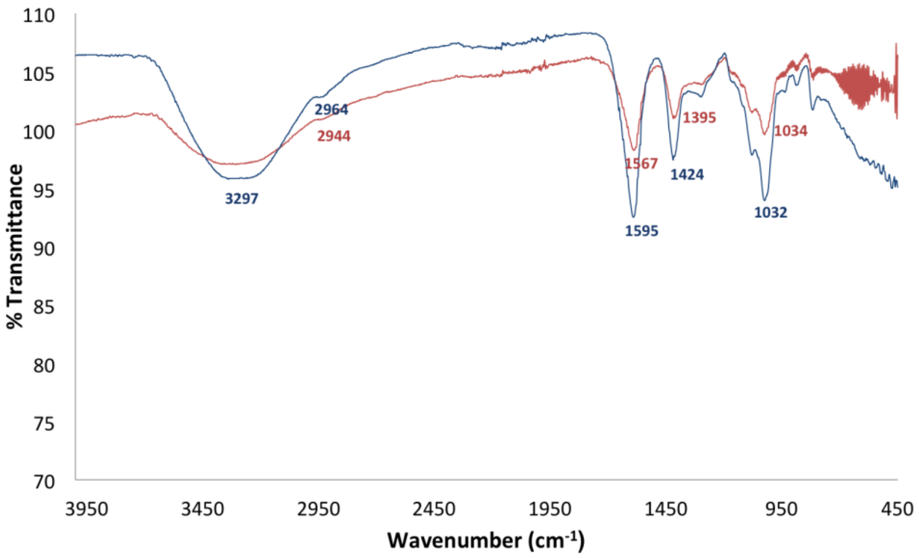

Figure S49. IR spectra of alginate gel filaments (0.8% wt/vol) with (red line) and without (blue line) AuNPs.

S4.5 Thermal stability studies

All the gels for T_{gel} determination were prepared as described in Section S4.1 in 7 mL vials (diameter: 2 cm, height: 6 cm) and loaded with $AuCl_3$ (5, 10 and 20 mM, 1 mL). The gel filaments for T_{gel} determination were prepared by dissolving DBS-CONHNH$_2$ (1.5, 3.0 and 4.5 % wt/vol) in DMSO and injecting 50 μL of the solution into a water bath with a syringe pump at a 3.4 μL/min flow rate (23G blunt tip needle). The filaments were isolated, transferred into a vial and then loaded with $AuCl_3$ (5, 10 and 20 mM, 1 mL). After 24 hours, the supernatant was removed. All gels were analysed in a high precision thermoregulated oil bath with an initial temperature of 25°C. The temperature was increased by 1°C/ min until 100°C. Every minute the gels were visually checked and T_{gel} was considered as the temperature at which the filaments showed visible signs of degradation. These experiments were performed in triplicate to ensure reproducibility and the average is reported. Errors are estimated at ±2°C.

Table S6. T_{gel} values of gels with AuNPs formed by individual gelators and the DBS-$CONHNH_2$/alginate hybrid gel.

Gel (1 mL total volume)	Loading of DBS-$CONHNH_2$ (wt/vol)	Loading of Alginate (wt/vol)	$AuCl_3$ concentration (mM)	T_{gel}
DBS-$CONHNH_2$	0.4%	-	-	86 °C
DBS-$CONHNH_2$	0.4%	-	5 mM	>100 °C
DBS-$CONHNH_2$	0.4%	-	10 mM	>100 °C
DBS-$CONHNH_2$	0.4%	-	20 mM	>100°C
Alginate	-	0.8%	-	>100 °C
Alginate	-	0.8%	5 mM	>100 °C
Alginate	-	0.8%	10 mM	>100 °C
Alginate	-	0.8%	20 mM	>100 °C
DBS-$CONHNH_2$/alginate hybrid gel	0.3%	0.5%	-	>100 °C
DBS-$CONHNH_2$/alginate hybrid gel	0.3%	0.5%	5 mM	>100 °C
DBS-$CONHNH_2$/alginate hybrid gel	0.3%	0.5%	10 mM	>100 °C
DBS-$CONHNH_2$/alginate hybrid gel	0.3%	0.5%	20 mM	>100 °C
DBS-$CONHNH_2$ filament	1.5%	-	-	88 °C
DBS-$CONHNH_2$ filament	1.5%	-	5 mM	93 °C
DBS-$CONHNH_2$ filament	1.5%	-	10 mM	93 °C
DBS-$CONHNH_2$ filament	1.5%	-	20 mM	95 °C
DBS-$CONHNH_2$ filament	3.0%	-	-	93 °C
DBS-$CONHNH_2$ filament	3.0%	-	5 mM	95 °C
DBS-$CONHNH_2$ filament	3.0%	-	10 mM	94 °C
DBS-$CONHNH_2$ filament	3.0%	-	20 mM	>100 °C
DBS-$CONHNH_2$ filament	4.5%	-	-	>100 °C
DBS-$CONHNH_2$ filament	4.5%	-	5 mM	>100 °C
DBS-$CONHNH_2$ filament	4.5%	-	10 mM	>100 °C
DBS-$CONHNH_2$ filament	4.5%	-	20 mM	>100 °C

S4.6 Rheology

Gel samples for rheology were prepared as described in Section S4.1 using bottomless vials as templates to obtain the intended gel dimensions. Gels loaded with AuNPs were washed with water and then treated with 1 mL of a 5 or 10 mM $AuCl_3$ solution (containing respectively 5.0 or 10.0 μmoles of Au (III)) for 24 hours. After 24 hours, the supernatant was removed and the mechanical properties of the gels were analysed. The measurements were carried out at 25°C using a 20 mm parallel plate and a gap of 2 mm. To avoid solvent evaporation and keep the sample hydrated, a solvent trap was used, and the internal atmosphere was kept saturated. Amplitude sweep experiments were performed in the range of 0.05-100% strain at a 1 Hz frequency to identify the linear viscoelastic region. Frequency sweep experiments were performed between 0.1 and 100 Hz using a shear strain of 0.25%. The measurements were repeated three times to ensure reproducibility and the average data are shown.

Table S7. Rheological data as determined using oscillatory rheometry with a parallel plate geometry, for DBS-CONHH$_2$, DBS-CONHNH$_2$/alginate gels and calcium alginate gels incorporating AuNPs. Loadings are given in wt/vol, and the G'/G" crossover points refer to the % shear strain at which G"=G'.

Gel	Loading of LMWG	Loading of Alginate	Total Loading	Concentration of $AuCl_3$ (1 mL) added	G' (Pa)	G" (Pa)	G'/G" Crossover
DBS-CONHNH$_2$	0.4%	-	0.4%	-	786	38	20.0%
DBS-CONHNH$_2$	0.4%	-	0.4%	5 mM	758	42	10.0%
DBS-CONHNH$_2$	0.4%	-	0.4%	10 mM	634	49	25.1%
Hybrid	0.3%	0.5%	0.8%	-	8260	940	6.9%
Hybrid	0.3%	0.5%	0.8%	5 mM	8870	1040	3.1%
Hybrid	0.3%	0.5%	0.8%	10 mM	16100	1710	8.0%
Alginate	-	0.8%	0.8%	-	2570	551	2.3%
Alginate	-	0.8%	0.8%	5 mM	6030	1010	10.1%
Alginate	-	0.8%	0.8%	10 mM	7390	1220	13.8%

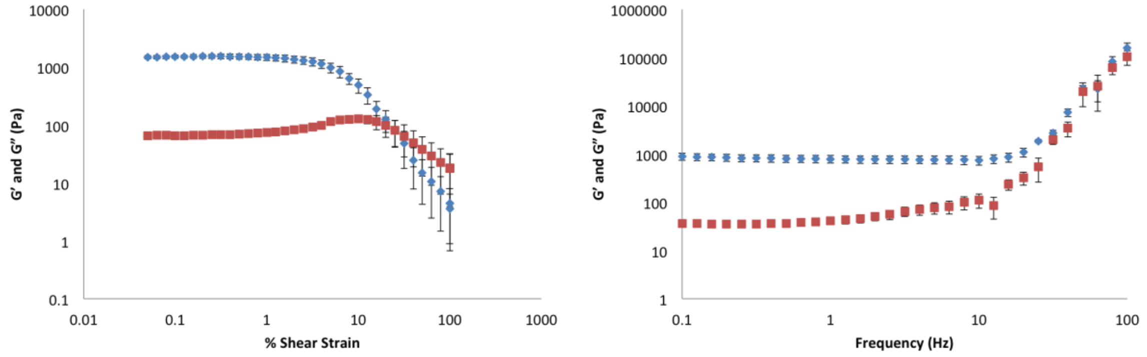

Figure S50. Elastic (G', blue circles) and viscous (G", red circles) moduli of DBS-CONHNH$_2$ hydrogel (0.4% wt/vol) with increasing shear strain (left) and frequency (right).

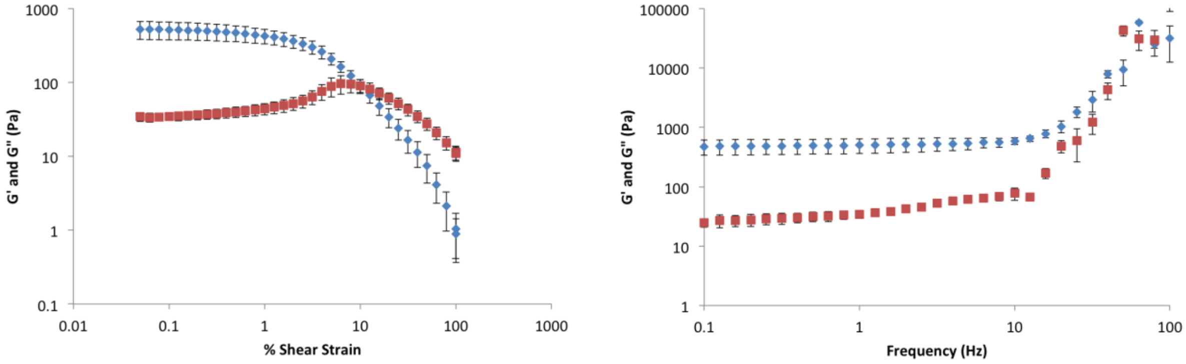

Figure S51. Elastic (G', blue circles) and viscous (G", red circles) moduli of DBS-CONHNH$_2$ hydrogel (0.4% wt/vol - loaded with 1 mL $AuCl_3$ 5 mM) with increasing shear strain (left) and frequency (right).

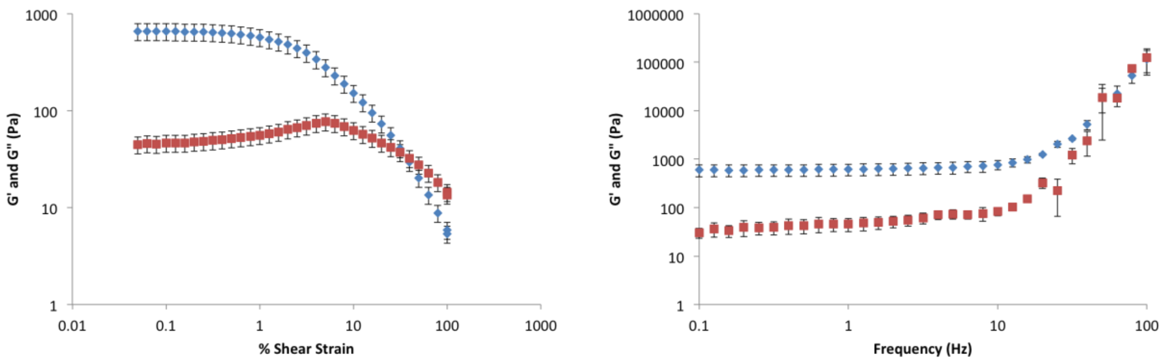

Figure S52. Elastic (G’, blue circles) and viscous (G”, red circles) moduli of DBS-CONHNH$_2$ hydrogel (0.4% wt/vol - loaded with 1 mL AuCl$_3$ 10 mM) with increasing shear strain (left) and frequency (right).

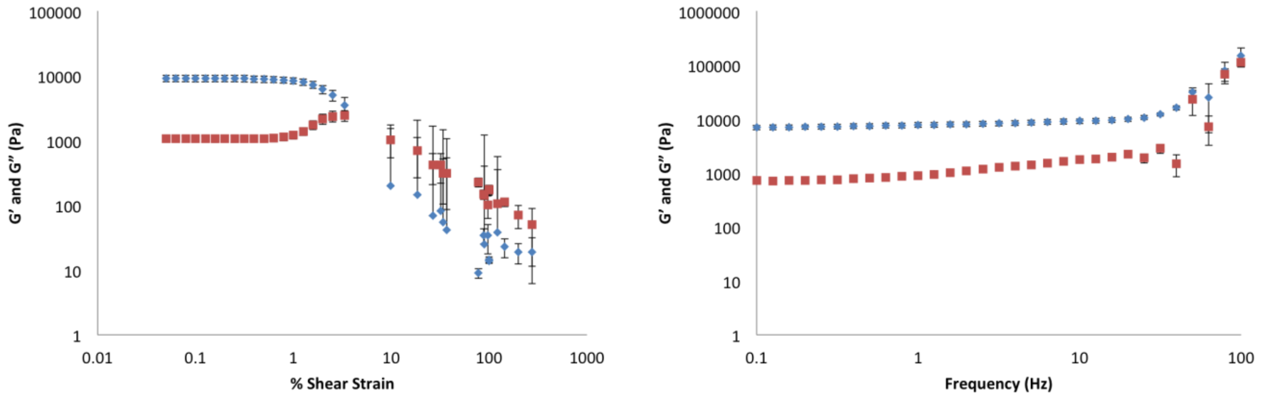

Figure S53. Elastic (G’, blue circles) and viscous (G”, red circles) moduli of DBS-CONHNH$_2$/alginate hydrogel (0.3% wt/vol DBS-CONHNH$_2$ and 0.5% wt/vol alginate) with increasing shear strain (left) and frequency (right).

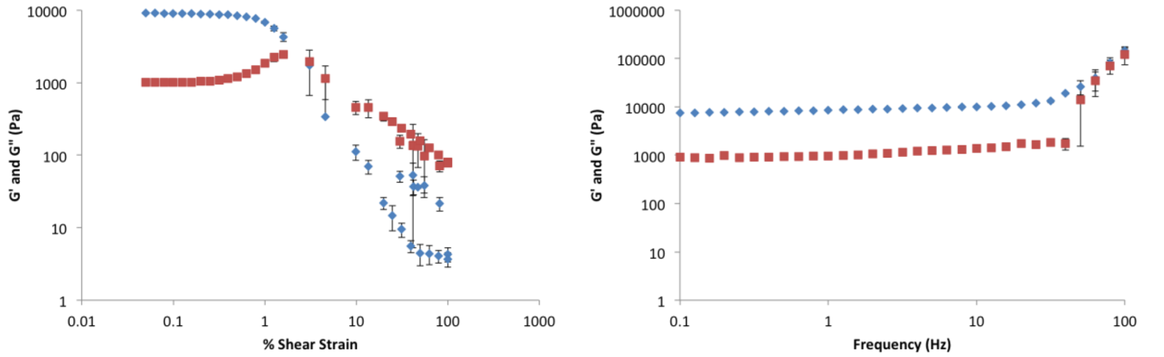

Figure S54. Elastic (G’, blue circles) and viscous (G”, red circles) moduli of DBS-CONHNH$_2$/alginate hydrogel (0.3% wt/vol DBS-CONHNH$_2$ and 0.5% wt/vol alginate - loaded with 1 mL AuCl$_3$ 5 mM) with increasing shear strain (left) and frequency (right).

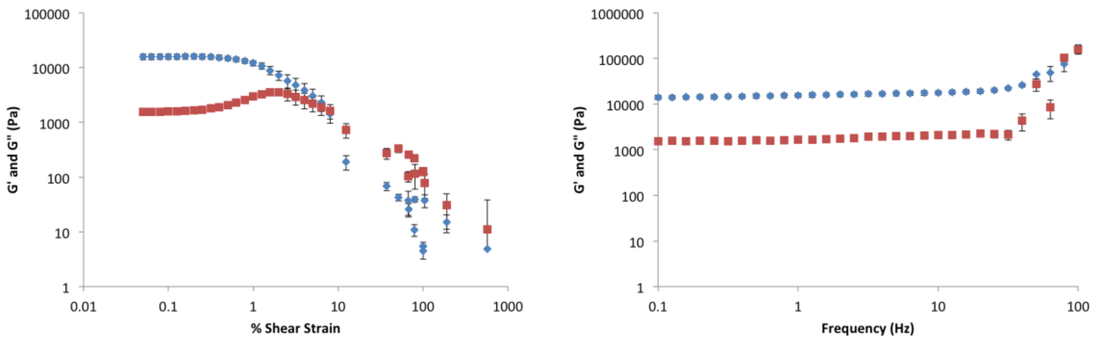

Figure S55. Elastic (G', blue circles) and viscous (G", red circles) moduli of DBS-CONHNH$_2$/alginate hydrogel (0.3% wt/vol DBS-CONHNH$_2$ and 0.5% wt/vol alginate - loaded with 1 mL AuCl$_3$ 10 mM) with increasing shear strain (left) and frequency (right).

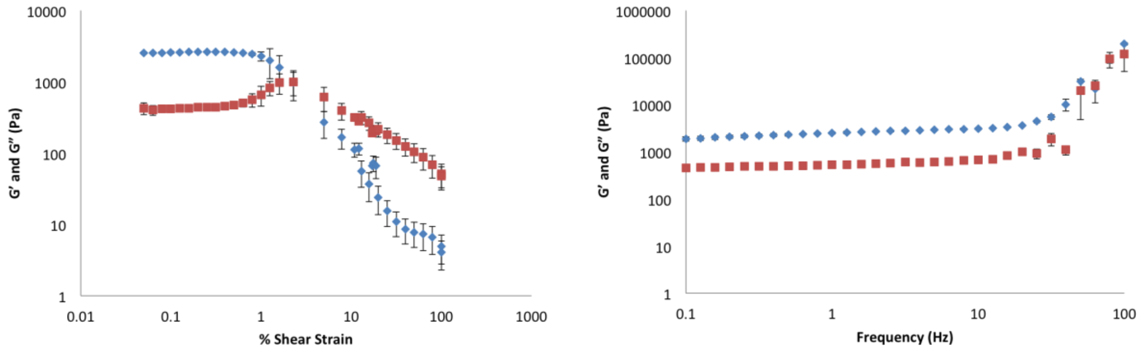

Figure S56. Elastic (G', blue circles) and viscous (G", red circles) moduli of alginate hydrogel (0.8% wt/vol) with increasing shear strain (left) and frequency (right).

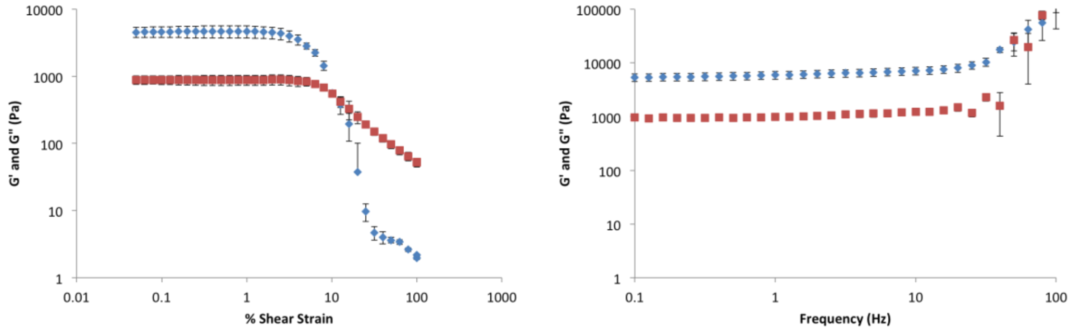

Figure S57. Elastic (G', blue circles) and viscous (G", red circles) moduli of alginate hydrogel (0.8% wt/vol - loaded with 1 mL AuCl$_3$ 5 mM) with increasing shear strain (left) and frequency (right).

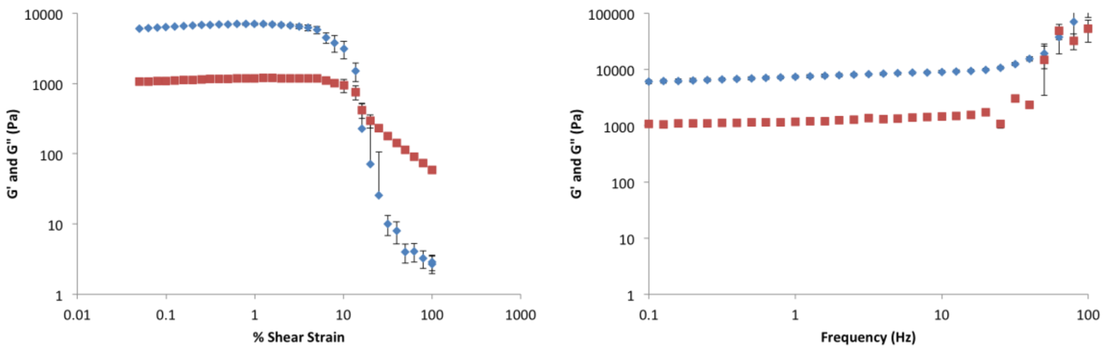

Figure S58. Elastic (G', blue circles) and viscous (G", red circles) moduli of alginate hydrogel (0.8% wt/vol - loaded with 1 mL $AuCl_3$ 10 mM) with increasing shear strain (left) and frequency (right).

S5 Biological studies

S5.1 Cell line (Y201 immortalized human mesenchymal stem cells – MSCs).

Y201 MSCs were grown in a T175 flask with Dulbecco's Modified Eagle's Medium (DMEM) with fetal bovin serum (FBS - 10%) and penicillin/streptomycin (P/S - 1%). To obtain the cells, the medium was removed from the flask and the cells washed with Dulbecco's phosphate buffer saline solution (11 mL). Trypsin/EDTA (2 mL) was then added and the cells were incubated at 37°C for approximately five mins. When cell detachment was observed by optical microscopy, trypsin was neutralised with 9 mL DMEM (10% FBS, 1% P/S). The cells were then transferred in a tube and isolated by centrifugation. After centrifugation, the supernatant was removed and the cell pellet was dispersed in 5 mL DMEM (10% FBS, 1% P/S). Cell count was performed using a Countess Automated Cell Counter (Thermo Fisher) on a 10 μL aliquot of a stock solution obtained by mixing 20 μL of cell suspension with 20 μL of trypan blue.

S5.2 Cytotoxicity assay

S5.2.1 Gel preparation. DBS-CONHNH$_2$/alginate (0.3% wt/vol DBS-CONHNH$_2$ and 0.5% wt/vol alginate) and alginate gels (0.8% wt/vol) for cytotoxicity assays were prepared in triplicate in a 48-well plate (300 μL volume) and cross-linked by addition of an equal volume of $CaCl_2$ (5% wt/vol), as described in Section S4.1. The *in situ* formation of Au NPs was induced by addition of an $AuCl_3$ solution (300 μL – 1.0 mM or 10.0 mM) on top of the gels. The gels were left undisturbed for 72 hours, subsequently washed with Dulbecco's Modified Eagle's Medium (DMEM - 400 μL) multiple times and then transferred in the middle of a 6-well plate.

DBS-CONHNH$_2$ gels were directly prepared in triplicate in a 6-well plate (300 μL volume; 0.3% wt/vol), using small bottomless vials (c.a. 1 cm diameter). Once the gels were formed, the vials were removed, leaving self-supporting gels in the middle of each well.

S5.2.2 Plate seeding. The cells (100000/well) were seeded on the bottom of the wells around the gels in the 6-well plates and covered with DMEM (10% FBS, 1% P/S - 2 [illegible]L).

S5.2.3 Crystal violet staining. After 48 hours, the DMEM was removed and each well was washed with PBS (1 mL). A crystal violet methanol solution (1 mL) was added to each well and the plates were left undisturbed for 20 mins. After 20 mins, the stain was collected and the plates were washed multiple times in a distilled water bath and then left to dry. Plates were imaged with an Epson PhotoScanner.

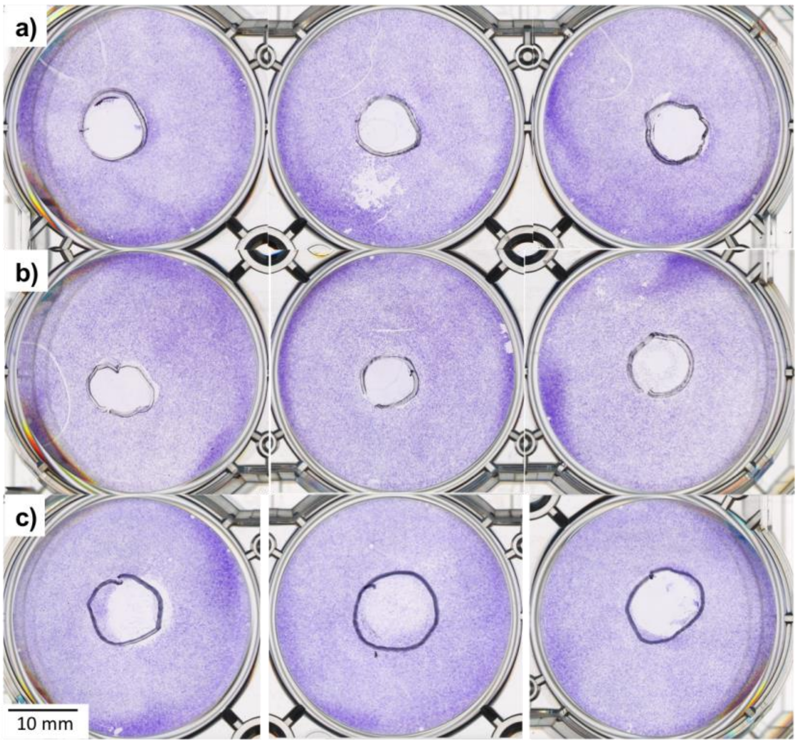

Figure S59. Scanned images of the cytotoxicity assay. Control gels without AuNPs. (a) DBS-CONHNH$_2$/alginate hybrid gels, (b) alginate gels, (c) DBS-CONHNH$_2$ gels. Scale bar: 10 mm.

Table S8. Zone of inhibition of cell growth around gels placed in the middle of a 6-well plate.

Gel	Loading of LMWG	Loading of Alginate	Total Loading	Concentration of $AuCl_3$ added (300 μL)	Concentration of $AgNO_3$ added (300 μL)	Zone of inhibition (average of 3 gels)	Standard deviation
DBS-CONHNH$_2$	0.3%	-	0.3%	-	-	0 mm	+/- 0 mm
Hybrid	0.3%	0.5%	0.8%	-	-	0 mm	+/- 0 mm
Hybrid	0.3%	0.5%	0.8%	1 mM	-	0 mm	+/- 0 mm
Hybrid	0.3%	0.5%	0.8%	10 mM	-	0 mm	+/- 0 mm
Alginate	-	0.8%	0.8%	-	-	0 mm	+/- 0 mm
Alginate	-	0.8%	0.8%	1 mM	-	0 mm	+/- 0 mm
Alginate	-	0.8%	0.8%	10 mM	-	0 mm	+/- 0 mm

S5.3 Viability assay

S5.3.1 Gel preparation in 96-well plates and plate seeding. Gels were prepared as described in Section S4.1 in 96-well plates in 75 μL volume. The *in situ* formation of Au NPs was induced by addition of an $AuCl_3$ solution (75 μL – 0.625, 1.25, 2.5, 5.0, and 10.0 mM) on top of the gels. The gels were left undisturbed for 72 hours. After this time, the supernatant was removed and the gels were washed multiple times with DMEM (10% FBS, 1% P/S - 200 μL). After the last wash, the gels were soaked with DMEM (10% FBS, 1% P/S - 100 μL) and the cells (25000/well) were seeded and covered with further DMEM (10% FBS, 1% P/S - 100 μL).

S5.3.2 Alamar Blue viability assay. Cell viability was measured at different time points using the Alamar Blue viability assay (*Thermo Fisher Scientific*). The cell culture medium was removed from each well and a

10% solution of Alamar Blue in DMEM (100 μL) was added. The plates were incubated at 37°C for 4 hours. After this time, 20 μL aliquots were taken from each well and diluted with DMEM (180 μL) in a new 96 well plate. Fluorescence was then measured with a fluorimeter (excitation 530-560 nm and emission 590 nm). This experiment was performed in sextuplicate and average values are reported with the error bars representing standard error. Control experiments with no cells were performed for each gel type.
Statistical analysis was performed using Graphpad Prism version 9.0.2 for Windows (GraphPad Software, San Diego, California USA). Data was tested for normality using Shapiro Wilk's test and transformed using y=log(y). A 2-way ANOVA with Geisser Greenhouse correction with Tukey's post hoc comparison was performed on all log transformed data to evaluate significant differences. Outcomes are displayed as mean +/- SEM with significant differences indicated from analysis of log transformed data (****$p<0.000$, ***$p<0.001$, **$p<0.01$, *$p<0.05$).

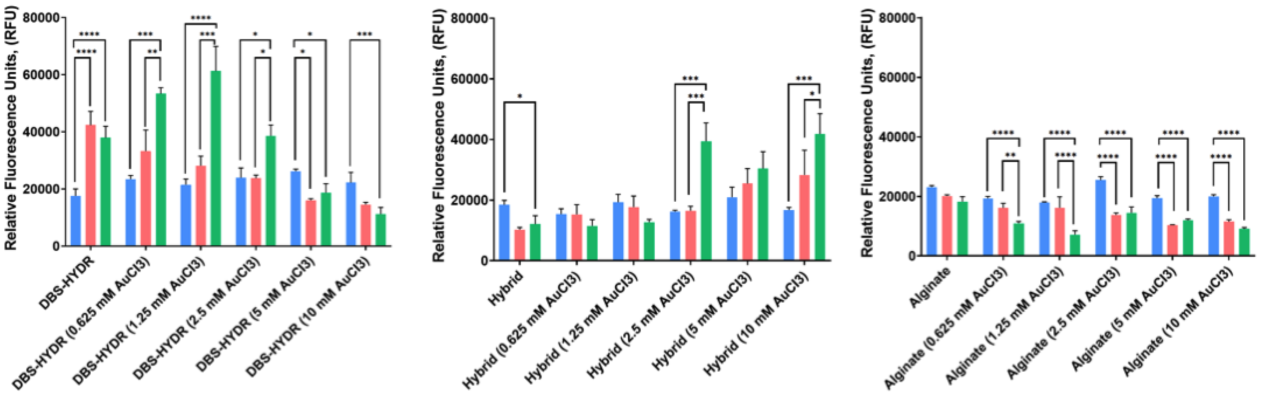

Figure S60. Alamar blue assay results for gels loaded with different $AuCl_3$ concentrations (N=6, mean reported, error bars represent standard error, DBS-HYDR = DBS-$CONHNH_2$

S6 References

[1] B. O. Okesola and D. K. Smith, *Chem. Commun.*, 2013, **49**, 11164-11166.
[2] D. J. Cornwell, B. O. Okesola and D. K. Smith, *Soft Matter*, 2013, **9**, 8730-8736.
